# Meta-Analysis of the Therapeutic Effects of Stem Cell-Derived Extracellular Vesicles in Rodent Models of Hemorrhagic Stroke

**DOI:** 10.1155/2024/3390446

**Published:** 2024-06-27

**Authors:** Conglin Wang, Bo Yan, Pan Liao, Fanglian Chen, Ping Lei

**Affiliations:** ^1^ Department of Geriatrics Tianjin Medical University General Hospital, Tianjin 300052, China; ^2^ School of Medicine Nankai University, Tianjin 300071, China; ^3^ Department of Neurology Xuanwu Hospital Capital Medical University, Beijing 100053, China

## Abstract

**Background:**

Stem cell-derived extracellular vesicles (SCEVs) have emerged as a potential therapy for hemorrhagic stroke. However, their effects are not fully understood. The aim of this study was to comprehensively evaluate the effects of SCEVs therapy in rodent models of hemorrhagic stroke, including subarachnoid hemorrhage (SAH) and intracerebral hemorrhage (ICH).

**Materials and Methods:**

We conducted a comprehensive search of PubMed, EMBASE, and Web of Science until May 2023 to identify studies investigating the effects of SCEVs therapy in rodent models of ICH. The functional outcomes were assessed using neurobehavioral scores. Standardized mean differences (SMDs) and confidence intervals (CIs) were calculated using a random-effects model. Three authors independently screened the articles based on inclusion and exclusion criteria. All statistical analyses were performed using Revman 5.3 and Stata 17.0.

**Results:**

Twelve studies published between 2018 and 2023 met the inclusion criteria. Our results showed that SCEVs therapy improved neurobehavioral scores in the rodent SAH model (SMD = −3.49, 95% CI: −4.23 to −2.75; *p* < 0.001). Additionally, SCEVs therapy improved the chronic neurobehavioral scores of the rodent ICH model (SMD = 2.38, 95% CI: 0.36–4.40; *p*=0.02) but did not have a significant impact on neurobehavioral scores in the acute and subacute phases. Significant heterogeneity was observed among the studies, and further stratification and sensitivity analyses failed to identify the source of heterogeneity.

**Conclusions:**

Our findings suggest that SCEVs therapy may improve neurofunctional behavior after hemorrhagic stroke and provide important insights into the design of preclinical trials.

## 1. Introduction

Hemorrhagic stroke, comprising intracerebral hemorrhage (ICH) and subarachnoid hemorrhage (SAH), is a critical medical condition with significant global mortality and morbidity rates [1]. It occurs when blood vessels rupture in the brain, leading to blood accumulation and subsequent damage to surrounding neural tissue [2]. Despite advancements in medical care, managing hemorrhagic stroke remains challenging because of the limited availability of effective treatments and the absence of definitive therapies [3]. The current standard of care includes supportive measures, surgical interventions, and addressing associated complications. However, these approaches often fail to address the underlying pathophysiological mechanisms that contributing to secondary brain injury and poor outcomes [4]. Consequently, there is an urgent need to explore novel therapeutic strategies to facilitate brain tissue repair, reduce inflammation, and enhance neurological recovery [5, 6].

In recent years, stem cell-based therapies have shown promise in the treatment of various neurological disorders, including hemorrhagic stroke [7, 8]. Stem cells possess unique abilities to self-renew and differentiate into various cell types, making them potential candidates for repairing damaged brain tissue [9, 10]. However, direct stem cell transplantation is fraught with challenges, such as stem cell survival, tumorigenicity, and ethical concerns [8]. A promising alternative is the use of extracellular vesicles (EVs) [11]. Stem cell-derived extracellular vesicles (SCEVs) are small membrane-bound vesicles secreted by cells that contain bioactive molecules, such as proteins, lipids, and nucleic acids [12, 13]. They play a crucial role in intercellular communication by transferring their cargo to recipient cells, thereby influencing cellular functions to promote tissue regeneration [14]. SCEVs carry a range of bioactive molecules derived from parent stem cells, including growth factors, cytokines, and genetic material, which can benefit damaged brain tissues [15, 16]. Moreover, SCEVs offer advantages over direct stem cell transplantation, such as lower immunogenicity, reduced tumor formation risk, and easier storage and administration [17].

SCEVs have been tested as a safe cell-free therapy for numerous diseases, including spinal cord injury and neural damage [18, 19]. Previous research has demonstrated that SCEVs can exhibit therapeutic effects on their own. For instance, adipose-derived SCEVs can alleviate inflammation and oxidative stress by regulating the Nrf2/HO-1 axis in macrophages [20]; EVs derived from mesenchymal stem cells (MSCs) can promote angiogenesis [21]. Additionally, SCEVs can serve as carriers for delivering drugs. For example, SCEVs overexpressing miRNA-21 can regulate the NF-*κ*B pathway, thereby protecting neural cells [22]. Duan et al. [2] injected miR-146a-5p-enriched SCEVs, which reduced neuronal apoptosis and inflammation after ICH, thereby improving impaired neural function. However, the therapeutic efficacy of SCEVs in hemorrhagic stroke requires further elucidation, necessitating a comprehensive analysis of the existing literature. Thus, the primary objective of this meta-analysis was to evaluate the treatment effects of SCEVs on hemorrhagic stroke. Through a systematic review and synthesis of the available evidence, we aim to provide a comprehensive assessment of the therapeutic potential, safety, and optimal administration protocols of SCEV in the treatment of hemorrhagic stroke.

## 2. Materials and Methods

### 2.1. Data Sources and Search Strategy

The researchers conducted a systematic literature search using three databases—MEDLINE, EMBASE, and Web of Science-to screen for targeted studies (all until May 15, 2023). The detailed search strategy is shown in *Supplementary table 1*. The reference lists of the included studies were also searched to identify other relevant articles.

### 2.2. Inclusion and Exclusion Criteria

Studies were included if they met the following criteria: (1) ICH and SAH models were induced in rodent animals; (2) the effect of unmodified stem cells or SCEVs was tested in at least one experimental group; (3) studies provided adequate data on neurobehavioral scores; (4) experimental studies were presented as original research and published in peer-reviewed journals; (5) studies were published in English; and (6) consisted of randomized or non-randomized controlled animal trials.

The exclusion criteria were as follows: (1) studies that did not include in vivo testing; (2) the outcome did not include the neurobehavioral scores; (3) studies that were published as clinical research, reviews, or conference abstracts; (4) use of other animals (dogs, monkeys or others) for hemorrhagic stroke model construction; (5) non-English articles; and (6) unpublished data.

### 2.3. Study Selection

Duplicate articles were automatically excluded from EndNote, and the remaining studies were manually selected by two independent researchers. Any disagreements were resolved through discussions with a third reviewer. The titles and abstracts of relevant articles were reviewed to identify eligible papers. Full-text articles were obtained and thoroughly reviewed for final eligibility based on the inclusion and exclusion criteria. The excluded articles and reasons for exclusion are shown in *Supplementary table 1*. We also read the relevant reviews containing potentially eligible articles and summarized the relevant articles (*Supplementary table 1*). The characteristics and quality assessments of other studies on the treatment of ICH with SCEVs are summarized in *Supplementary table 1*.

### 2.4. Data Extraction

Three researchers (Conglin Wang, Yan Bo, and Pan Liao) independently extracted the following information from each study: lead author, publication year, country, species(sex), weight/year, anesthetic, method of stroke, number of treatment/control animals, stem cell species (extracellular vesicles/exosomes), compatible stem cells dose, stem cell route, time of administration, assessment time, functional outcome (neurobehavioral scores), and potential mechanism.

We collected data on the mean and standard deviation (SD) of neurobehavioral scores. If the SD was not reported, it was calculated by multiplying the standard error (SE) by the square root of the sample size. If the study had more than two groups designed and permitted multiple comparisons, we extracted only the information and data of interest reported in the original articles. If only graphs were available, values were calculated from images using GetData Graph Digitizer software.

### 2.5. Quality Assessment

We recorded these data with reference to the Collaborative Approach to Meta-Analysis and Review of Animal Data from Experimental Stroke (CAMARADES) [23]: (1) peer-reviewed publication; (2) statement of control of temperature; (3) random allocation to treatment or control; (4) blinded induction; (5) blinded assessment of outcome; (6) use of anesthetic without significant intrinsic neuroprotective activity (such as ketamine); (7) use of animals with comorbidities; (8) sample size calculation; (9) compliance with animal welfare regulations; and (10) statement of potential conflict of interests. We defined studies that scored <5 points as low quality and those that scored ≥5 points as high quality.

### 2.6. Statistical Analysis

The combined effect size was calculated as the standardized mean difference (SMD) with a 95% confidence interval (95% CI) between the treatment group and control groups. A forest plot was generated to display the SMD and 95% CI of each study and the pooled the mean difference was by combining all studies. A random-effects model was used to pool the data, and statistical heterogeneity between summary data was evaluated using the *I*^2^ statistic. A sensitivity analysis was performed by excluding low-quality studies. Sensitivity and stratification analyses were performed to identify the sources of heterogeneity and to investigate other potential confounding factors [24]. A funnel plot was used to check for publication bias, the asymmetry of which was evaluated using Egger's test and the trim-and-fill method [25].

All the meta-analyses were performed using Revman version 5.3 (Cochrane Collaboration) and Stata 17 (StataCorp, College Station, TX, USA). All tests were two-tailed, and *p* < 0.05 was considered statistically significant.

## 3. Results

### 3.1. Study Inclusion

**Figure 1:**
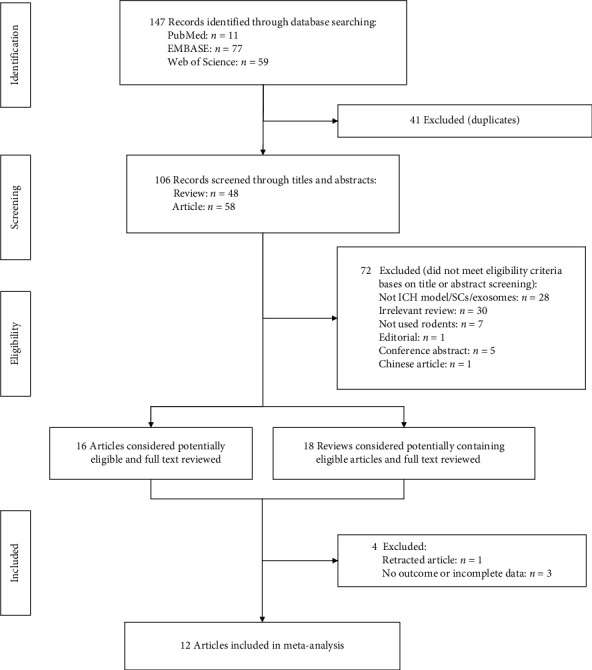
Flowchart of the enrolled studies on SCEVs therapy in rodents with hemorrhagic stroke.

This study was conducted and reported in compliance with the Preferred Reporting Items for Systematic Reviews and Meta-Analyses (PRISMA) guidelines [26]. The study selection process is illustrated in Figure 1. A preliminary literature search identified 147 potential studies: 11 records in PubMed, 77 records in Embase, and 59 records in Web of Science. After review and exclusion, 16 full-text articles remained and were evaluated for inclusion. At the same time, by evaluating the eligibility for inclusion, we left 18 potential reviews, which we attempted to supplement the literature with a review of the full texts of the reviews and references. However, all the articles included in the reviews were all duplicates (*Supplementary table 1*). After careful full-text reading of the articles, we excluded four articles for the following reasons: no results or incomplete data (*n* = 3), and articles were withdrawn. Our study included 12 articles published between 2018 and 2023 that met the inclusion criteria [27, 28, 29, 30, 31, 32, 33, 34, 35, 36, 37, 38].

### 3.2. Study Characteristics

Table 1 presents an overview of the included studies. Five studies focused on ICH, whereas seven studies examined SAH. Mice and rats were used for all animal models. Eleven studies used various sources of MSCs, and one study used adipose-derived stem cells (ADSCs). Regarding the administration of EVs, three ICH studies employed tail vein injection, and two studies used ventricle injection. The neurological scores used in this study included the modified Morris water maze (mMWM), modified neurological severity score (mNSS), odor recognition, negative geotaxis, and rotarod tests. The evaluation indices for neural function in the SAH model were based on the modified Garcia scoring system. Motor function evaluation time for searching for cohorts occurred exclusively in the acute stage (1–3 days) in the SAH model, whereas in the ICH model, it was divided into the acute stage (1–3 days), subacute stage (7–14 days), and chronic stage (28–35 days).

### 3.3. Study Quality

The included studies exhibited high methodological quality, with quality scores ranging from 6 to 9 (mean = 7.33), exceeding the threshold of 5. All studies were published in peer-reviewed journals, randomized animals into treatment or control groups, used appropriate animal models, complied compliance with animal welfare regulations, and reported potential conflicts of interest. Further details regarding the quality indicators are provided in Table 2.

### 3.4. Meta-Analysis

**Figure 2:**
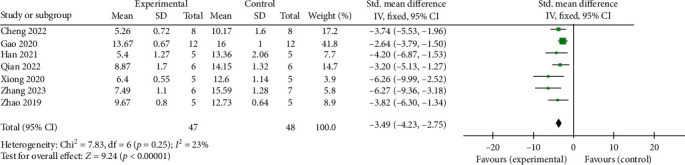
Forest plot showing the impact of SCEVs therapy on neurobehavioral scores in SAH, compared with controls. 95% CI: 95% confidence interval.

**Figure 3:**
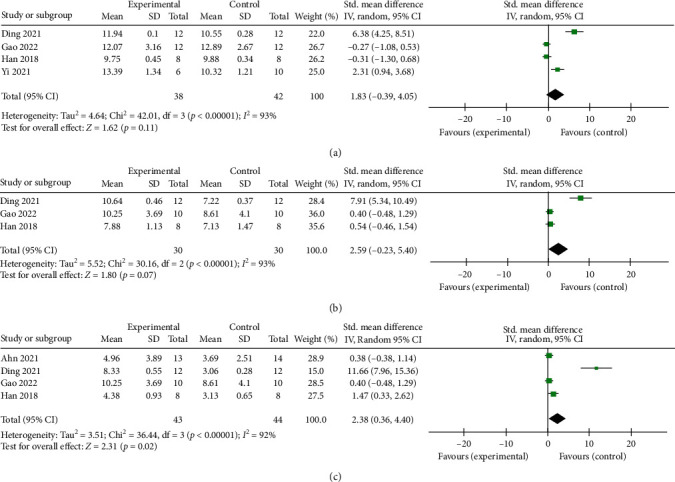
Forest plot showing the impact of SCEVs therapy on neurobehavioral scores in ICH, compared with controls: (a) acute stage (1−3 days); (b) subacute stage (7−14 days); (c) chronic stage (28−35 days). 95% CI: 95% confidence interval.

Neurobehavioral scores were reported in all studies. The meta-analysis demonstrated that SCEVs significantly improved neurobehavioral outcomes in the rodent SAH model compared to controls, SMD = −3.49 (95% CI: −4.23, −2.75; *p* < 0.001) (Figure 2). SCEVs therapy improved the chronic neurobehavioral scores in rodent ICH model, SMD = 2.38 (95% CI: 0.36, 4.40; *p*=0.02), but did not improve neurobehavioral scores in the acute and subacute phases, SMD = 1.83 (95% CI: −0.39, 4.05; *p*=0.11); SMD = 2.59 (95% CI: −0.23, 5.40; *p*=0.07). There was statistically significant heterogeneity in neurobehavioral outcomes during the chronic ICH stage (*I*^2^ = 92%, *p* < 0.001) (Figure 3).

### 3.5. Sensitivity Analysis

**Figure 4:**
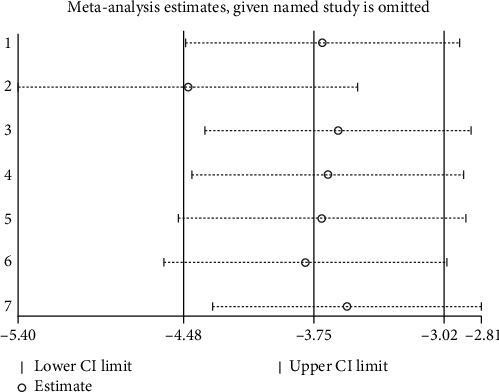
Sensitivity-analysis results of SAH.

**Figure 5:**
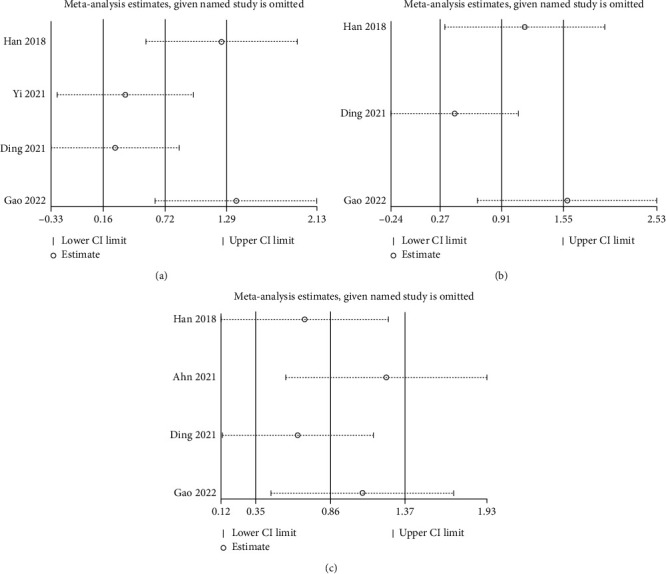
Sensitivity-analysis results of ICH: (a) acute stage (1−3 days); (b) subacute stage (7−14 days); (c) chronic stage (28−35 days).

To assess the robustness of the results, we performed a sensitivity analysis by sequentially omitting each study. None of the studies significantly influenced the pooled SMD of neurobehavioral outcomes (Figures 4 and 5).

### 3.6. Stratified Analysis

Details of the stratified analysis of the neurobehavioral scores for SAH and ICH are shown in Tables 3 and 4, respectively. For the neurobehavioral scores in SAH, we stratified the data by animal type, with no significant differences in the effect size estimates between rats and mice (*p*=0.54, *Supplementary figure 2*). Notably, after stratifying the data by narcotic drugs, studies using isoflurane showed higher effect sizes than other studies (*p*=0.09, *Supplementary figure 2*). The methods used to induce the SAH model did not differ significantly in effect size estimates (*p*=0.97, *Supplementary figure 2*). Stem cell type did not differ in terms of the estimated effect size (*p*=0.97, *Supplementary figure 2*). With regard to the route of administration, there was no difference in the estimate of effect size between modes of administration (*p*=0.13, *Supplementary figure 2*).

In the stratified analysis of the acute phase of ICH, we observed no significant differences in the estimated effect sizes when using different animal types (*p*=0.58, *Supplementary figure 2*). Studies employing pentobarbital exhibited higher effects compared to other studies (*p* < 0.001, *Supplementary figure 2*). Similarly, studies utilizing the collagenase model demonstrated a larger effect size than other studies (*p*=0.03, *Supplementary figure 2*). There were no significant differences in the estimated effect sizes of the SCEVs from different sources (*p*=0.73, *Supplementary figure 2*). In the subacute phase of ICH, stratified analysis revealed no significant differences in the estimated effect sizes using different animal types (*p*=0.30, *Supplementary figure 2*). Studies employing both pentobarbital and collagenase showed greater effects than other studies (*p* < 0.001, *Supplementary figure 2*). For the chronic phase of ICH, the stratified analysis indicated that studies using SD rats showed greater effects than other studies (*p*=0.06, *Supplementary figure 2*). Studies employing pentobarbital also demonstrated greater effects than other studies (*p* < 0.001, *Supplementary figure 2*). Furthermore, there were no significant differences in the estimated effect sizes when using different ICH models and SCEVs from different sources were used (*p* > 0.05, *Supplementary figure 2*, respectively).

### 3.7. Publication Bias

**Figure 6:**
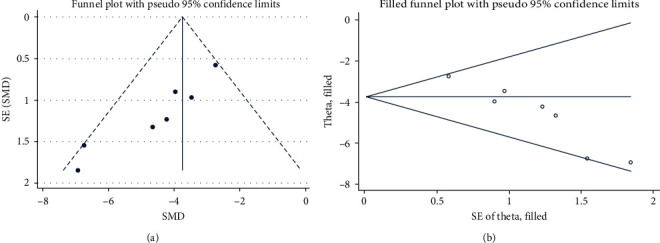
Publication-bias analysis results of SAH: (a) funnel plots for neurobehavioral scores; (b) trim-and-fill method was used to evaluate the missing studies in neurobehavioral scores.

We observed no significant publication bias in the neurobehavioral scores by visually examining the funnel plot of the SAH model (Figure 6(a)). However, after the Egger test was performed, a significant publication bias was found (*p*=0.001). We then recalculated the combined estimates using the trim-and-fill method and added the missing studies. However, the overall results did not change significantly (Figure 6(b)), indicating that there were no “missing” studies.

## 4. Discussion

This meta-analysis aimed to provide a comprehensive summary of the effects of SCEVs therapy in rodent models of hemorrhagic stroke. By analyzing 12 studies, we have identified the neuroprotective benefits of SCEVs in a preclinical rodent model of SAH and in the chronic phase of ICH. These findings have significant implications for human clinical trials that explore the therapeutic potential of SCEVs therapy. However, it is important to note that the limited number of studies highlights the need for additional research to further validate the neuroprotective effects of SCEVs therapy in experimental hemorrhagic stroke.

### 4.1. Selection and Administration of SCEVs

In our compilation of 15 studies on SCEVs for the treatment of hemorrhagic stroke, 93.3% of the studies used MSC-derived EVs, whereas 6.67% used ADSC-derived EVs. MSCs are a type of stem cells that possess self-renewal and multilineage differentiation potential and are widely distributed in the body, particularly in the bone marrow, adipose tissue, and placenta [39, 40]. MSCs have several advantages compared to other stem cells, such as ease of extraction, expansion in culture, and low immunogenicity [41]. Moreover, in the context of neurological therapy, MSCs can promote angiogenesis and remodeling by releasing various growth factors and cytokines, improving blood supply, enhancing nutrient and oxygen delivery to the brain tissue, and providing neuroprotection [42, 43]. A previous meta-analysis also provided information on the usefulness of MSCs in SAH [44]. In summary, MSCs have demonstrated potential as novel therapeutic agents for the treatment of hemorrhagic stroke.

However, the optimal application dosage of SCEVs for the treatment of hemorrhagic stroke remains unclear. Despite preliminary experiments and clinical studies, standardized methods to determine the optimal dosage are currently lacking [45]. Researchers mainly determine the dosage of EVs by assessing the protein content of the extracellular vesicles or based on the quantity of MSCs. It should be noted that the application dosage of EVs may be influenced by various factors, including the type and severity of the disease and the source and preparation method of the EVs, among others [46]. Furthermore, the lack of standardized methods for dosage determination poses a challenge for current research.

Regarding the delivery strategy of SCEVs in the treatment of hemorrhagic stroke, research has made some progress but is still in its early stages. Researchers have explored different routes of EVs administration [47]. Common routes of administration include intravenous and intraventricular injections. Different routes of administration may have varying effects on the EV-biodistribution, stability, and therapeutic efficacy. In our compilation of 15 studies on SCEVs for the treatment of hemorrhagic stroke, 80% used IV injection as the intervention method. Intranasal administration has also been used in other studies on EVs [48]. Intranasal administration of EVs allows them to enter the central nervous system through the nasal mucosa, cross the blood–brain barrier, and directly influence the central nervous system [49]. Additionally, intranasal administration is a noninvasive method that does not require surgery or injections, making it relatively simple, safe, and well-tolerated, reducing the discomfort and risks associated with treatment [50]. Further research is needed to determine the optimal administration strategies and ensure the safety and efficacy of the treatment.

### 4.2. Possible Mechanisms of SCEVs Therapy in Hemorrhagic Stroke

Although the neuroprotective effects of SCEVs therapy in ischemic stroke are widely accepted, its therapeutic potential in hemorrhagic stroke is only at the preliminary stage of exploration [51, 52]. The results of Ahn et al. [28] showed that MSC-derived exosomes were significantly attenuated IVH-induced TUNEL-positive apoptotic cell death, inflammatory response, oxidative stress, and severe increase in astrogliosis, whereas BDNF-siRNA-transfected MSCs derived exosomes abolished reduced brain myelination and neurogenesis. Yi et al. [29] suggested that EVs derived from ADSCs overexpressing miR-19b-3p exert neuroprotective effects by targeting the expression of the iron regulatory protein IRP2, which attenuates ICH-induced ferroptosis. Another study suggested that the improved recovery of neurobehavioral function after EVs treatment may be related to an increase in angiogenesis, white matter remodeling, vascular redistribution, and neurogenesis [27]. Bone marrow MSC-EVs carrying miR-183-5p repaired the HG-Hemin-BV2 cell inflammation by regulating the PDCD4/NLRP3 axis and improving the behavior and neuroinflammation following ICH [37]. Shen et al. [5] found that the administration of miR-133b-containing MSCs-EVs inhibited RhoA, activated the ERK1/2/CREB pathway, and ameliorated brain damage (including neuronal apoptosis and neurodegeneration) in rats after ICH. There has also been reported that miR-146a-5p-riched bone marrow MSCs-EVs could offer neuroprotection and functional improvement after ICH by reducing neuronal apoptosis and inflammation associated with the inhibition of microglial M1 polarization by downregulating the expression of IRAK1 and NFAT5 [2].

Zhao et al. [30] suggested that SCEVs could regulate early brain injury, neurological function, brain edema, and neuronal apoptosis after SAH. Gao et al. [32] suggested that MSC-EVs alleviate brain injury after SAH by inhibiting neuronal apoptosis and improving neurological behavior. Other experiments have suggested that small-molecule RNA carried by SCEVs plays a therapeutic role in alleviating the early brain damage caused by SAH [31, 33, 34, 35, 36].

SCEVs are involved in the regulation of pathophysiological processes after ICH, including neuronal apoptosis, inflammatory response, oxidative stress, changes in the number of astroglial proliferation, brain myelination, angiogenesis, white matter remodeling, neurogenesis, ferroptosis, and polarized responses in microglia.

### 4.3. Recommendations for Future Research on SCEVs

Meta-analyses of animal studies can often guide research and clinical practices. Preclinical meta-analyses could also be used to assess the safety of EV treatment in future clinical trials. To date, no clinical trials on SCEVs therapy for ICH have been conducted. However, EVs must undergo preclinical studies before they can be used in clinical research, and much work remains to be done. First, only a few animal studies have evaluated the therapeutic effects of SCEVs on ICH. We recommend that everyone actively participates in the research of this project and strives to promote the clinical translation of SCEVs. Second, in animal models of ICH, we observed that the vast majority of research subjects were rodents, which cannot mimic the physiological and pathological conditions of human ICH. We suggest that, in future studies, efforts should be made to establish more primate models while focusing on human SCEVs for more robust findings. Third, regarding the standardization of SCEVs. We suggest that future preclinical experiments should report the source of EVs, standardize the extraction and identification methods of EVs, increase follow-up time points, and use neurological function scores as prognostic indicators. More animal studies in clinical settings and clinical trials are needed to determine the therapeutic effects of SCEVs therapy in patients with ICH.

When SCEVs are used in clinical settings, the dosage and timing of their administration are often topics of concern. In different studies, differences in extraction methods may lead to the heterogeneity of results, and it is necessary to standardize EV extraction methods to unify EV dosing modes. Second, in most preclinical studies, SCEVs are administered within 1 hr of hemorrhagic stroke induction; however, in practice, most stroke patients receive formal treatment more than 1 hr after stroke. More animal studies and clinical trials are needed to determine the optimal timing of administration of SCEVs in patients with hemorrhagic stroke. In conclusion, meta-analyses of animal studies can often guide future research and clinical studies. Preclinical meta-analyses could also be used to assess the safety of EV treatment to design in future clinical trials. Owing to the beneficial effects of SCEVs therapy in animal models of ICH, clinical translation of SCEV therapy for the treatment of ICH is promising.

## 5. Conclusion

To our knowledge, this systematic review and meta-analysis is the first to investigate the effects of SCEVs on neuromotor function in hemorrhagic stroke animal models. Our analysis suggests that SCEVs therapy may enhance neurofunctional behavior posthemorrhagic stroke, offering valuable insights for the design of future preclinical trials.

## Figures and Tables

**Table 1:** .Characteristics of the included studies.

Author (year)	Country	Types	Species (gender)	Weight/year	Anesthetic	Molding method	No. of treated/controls animals	SC species	EVs dose	SC route	Time of administration	Assessment time	Neurobehavioral scores
Zhang et al. 2018 [22]	American	ICH	Adult Wistar rats (male)	270–300 g	NA	Autogenous blood	8/8	BM-MSCs	3 × 10^6^ cells	Tail vein	24 hr post-ICH	28 days	Modified Morris water maze (mMWM), modified neurological severity scores (mNSS) and odor recognition
Ahn et al. 2021 [28]	South Korea	ICH	New-born SD rats	Postnatal 4 day	Isoflurane	Maternal blood	14/13	Human umbilical cord MSC	20 *μ*g EVs	Intravenous	IVH within 24 hr	28 days	Negative geotaxis and rotarod tests
Yi et al. 2021 [29]	China	ICH	C57BL/6 mice	8–12 weeks	NA	Collagenase	10/10	Adipose-derived stem cells (ADSCs)	20 *μ*g EVs	Tail vein	2 hr post-ICH	2 days	mNSS
Ding et al. 2021 [37]	China	ICH	SD rats (male)	8–9 weeks	Pentobarbital (800 mg/kg)	Collagenase IV	12/12	Rat BM-MSCs	100 *μ*g EVs	Tail vein	24 hr post-ICH	1, 7, 28 days	Movement defect score and beam walking score
Gao et al. 2022 [38]	China	ICH	C57BL/6 mice	NA	3% isoflurane/air mixture	Autologous whole blood	12/12	Mice BM-MSCs	86 mg/kg	Intravenous	NA	1, 3, 7, 14, 35 days	Adhesive removal test and paw-fault test
Zhao et al. 2019 [30]	China	SAH	Sprague–Dawley (SD) rats	200–220 g	Pentobarbital	Double blood model	5/5	Human umbilical cord MSC	400 *μ*g EVs	Femoral vein	1 hr	1 day	Modified Garcia scoring system
Gao et al. 2020 [32]	China	SAH	SD rats	300–320 g	Chloral hydrate	Intravascular perforation SAH model	12/12	BM-MSCs	5–8 × 106 cells	i.v.	NA	2 days	Modified Garcia scoring system
Xiong et al. 2020 [31]	China	SAH	Sprague–Dawley rats	300–350 g	Isoflurane	Endovascular perforation	5/5	BM-MSCs	200 *μ*g EVs	Femoral vein	NA	1 day	Modified Garcia scoring system
Han et al. 2021 [33]	China	SAH	Sprague–Dawley rats	280–320 g, 7–8 weeks old	Isoflurane	Endovascular perforation	5/5	BM-MSCs	100 *μ*g EVs	Tail vein	10 min	1, 2 days	Modified Garcia scoring system
Cheng et al. 2022 [34]	China	SAH	SD rats	300−350 g	Isoflurane	Endovascular perforation	8/8	BM-MSCs	200 *μ*g EVs	Tail vein	1 hr	2 days	Modified Garcia scoring system
Qian et al. 2022 [35]	China	SAH	C57BL/6J mice	18–22 g, 8–10 weeks	Sodium pentobarbital	Endovascular perforation	6/6	BM-MSCs	200 *μ*l EVs	Femoral vein	1 hr	1 day	Modified Garcia scoring system
Zhang et al. 2023 [22]	China	SAH	SD rats	240–280 g	Isoflurane	Endovascular perforation	6/7	BM-MSCs	100 *μ*g EVs	Lateral ventricle	1 day	3 days	Modified Garcia scoring system

**Table 2:** Methodological quality of 12 studies included in the meta-analysis.

Author (year)	Types	Peer-reviewed publication	Statement of control of temperature	Random allocation to treatment or control	Blinded induction	Blinded assessment of outcome	Use of anesthetic without significant intrinsic neuroprotective activity	Suitable animal models	Sample size calculation	Compliance with animal welfare regulations	Statement of potential conflict of interests
Han et al. 2018 [27]	ICH	√	—	√	√	—	—	√	—	√	√
Ahn et al. 2021 [28]	ICH	√	√	√	√	—	√	√	—	√	√
Yi 2021 [29]	ICH	√	√	√	√	—	—	√	—	√	√
Ding et al. 2021 [37]	ICH	√	—	√	√	—	√	√	—	√	√
Gao et al. 2022 [38]	ICH	√	√	√	√	√	√	√	—	√	√
Zhao et al. 2019 [30]	SAH	√	√	√	—	—	—	√	—	√	√
Gao 2020 [32]	SAH	√	—	√	√	—	—	√	—	√	√
Xiong et al. 2020 [31]	SAH	√	—	√	√	√	√	√	—	√	√
Han et al. 2021 [33]	SAH	√	√	√	√	√	√	√	—	√	√
Cheng et al. 2022 [34]	SAH	√	√	√	√	—	√	√	—	√	√
Qian et al. 2022 [35]	SAH	√	—	√	—	√	—	√	—	√	√
Zhang et al. 2023 [22]	SAH	√	√	√	—	√	√	√	—	√	√

**Table 3:** Stratified meta-analysis of heterogeneity on neurobehavioral scores in SAH.

Categories	No. of studies	Pooled SMD (95% CI)	*p* value	Heterogeneity test			Between groups *p* value
				*Q statistics*	*I* ^2^	*p* value	
*Animal type*							0.54
Rat	6	−3.91 (−5.03, −2.79)	<0.001	7.73	35%	0.17	
Mice	1	−3.20 (−5.13, −1.27)	0.001	NA	NA	NA	
*Anesthetic type*							0.09
Pentobarbital	2	−3.43 (−4.96, −1.91)	<0.001	0.15	0%	0.70	
Chloral hydrate	1	−2.64 (−3.79, −1.50)	<0.001	NA	NA	NA	
Isoflurane	4	−4.55 (−5.81, −3.29)	<0.001	2.85	0%	0.42	
*Method of SAH*							0.97
Autogenous blood	1	−3.82 (−6.30, −1.34)	0.003	NA	NA	NA	
Endovascular perforation	6	−3.77 (−4.84, −2.70)	<0.001	7.76	36%	0.17	
*SCEVs type*							0.97
BM-MSC	6	−3.77 (−4.84, −2.70)	<0.001	7.76	36%	0.17	
UC-MSC	1	−3.82 (−6.30, −1.34)	0.003	NA	NA	NA	
*Delivery route*							0.13
Tail vein injection	2	−3.88 (−5.37, −2.40)	<0.001	0.08	0%	0.78	
Femoral vein injection	3	−3.84 (−5.27, −2.42)	<0.001	2.03	1%	0.36	
Lateral ventricle injection	1	−6.27 (−9.36, −3.18)	<0.001	NA	NA	NA	
IV	1	−2.64 (−3.79, −1.50)	<0.001	NA	NA	NA	

**Table 4:** Stratified meta-analysis of heterogeneity on neurobehavioral scores in ICH.

Categories	No. of studies	Pooled SMD (95% CI)	*p* value	Heterogeneity test			Between groups *p* value
				*Q statistics*	*I* ^2^	*p* value	
*Acute phase*							
*Animal type*							0.58
Rats	2	2.97 (−3.59, 9.52)	0.37	31.18	97%	0.3	
Mice	2	1.01 (−1.42, 3.44)	0.42	8.95	89%	0.003	
*Anesthetic type*							<0.001
Pentobarbital	1	6.38 (4.25, 8.51)	<0.001	NA	NA	NA	
Isoflurane	1	−0.17 (−1.05, 0.70)	0.7	NA	NA	NA	
NR	2	0.95 (−1.61, 3.52)	0.47	9.23	89%	0.002	
*Method of ICH*							0.03
Autogenous blood	2	−0.23 (−0.89, 0.42)	0.49	0.04	0%	0.84	
Collagenase	2	4.26 (0.27, 8.25)	0.04	9.95	90%	0.002	
*SCEVs type*							0.73
BM-MSC	3	1.75 (−1.07, 4.58)	0.22	33.86	94%	<0.001	
UC-MSC	1	2.31 (0.94, 3.68)	<0.001	NA	NA	NA	
*Subacute phase*							
*Animal type*							0.30
Rats	2	4.13 (−3.09, 11.35)	0.26	27.31	96%	<0.001	
Mice	1	0.29 (−0.59, 1.18)	0.51	NA	NA	NA	
*Anesthetic type*							<0.001
Pentobarbital	1	7.91 (5.34, 10.49)	<0.001	NA	NA	NA	
Isoflurane	1	0.29 (−0.59, 1.18)	0.51	NA	NA	NA	
NR	1	0.54 (−0.46, 1.54)	0.29	NA	NA	NA	
*Method of ICH*							<0.001
Autogenous blood	2	0.40 (−0.26, 1.06)	0.24	0.13	0%	0.72	
Collagenase	1	7.91 (5.34, 10.49)	<0.001	NA	NA	NA	
*Chronic phase*							
*Animal type*							0.06
Rats	3	3.69 (0.40, 6.99)	0.03	35.15	94%	<0.001	
Mice	1	0.40 (−0.48, 1.29)	0.37	NA	NA	NA	
*Anesthetic type*							<0.001
Pentobarbital	1	11.66 (7.96, 15.36)	<0.001	NA	NA	NA	
Isoflurane	1	0.40 (−0.48, 1.29)	0.37	NA	NA	NA	
NR	2	−0.84 (−0.22, 1.90)	0.12	2.43	59%	0.12	
*Method of ICH*							0.38
Autogenous blood	2	0.87 (−0.17, 1.92)	0.1	2.1	52%	0.15	
Collagenase	2	5.87 (−5.18, 16.92)	0.3	34.33	97%	<0.001	
*SCEVs type*							0.06
BM-MSC	3	3.75 (0.36, 7.14)	0.03	34.09	94%	<0.001	
UC-MSC	1	0.38 (−0.38, 1.14)	0.33	NA	NA	NA	

## Data Availability

The original contributions presented in the study are included in the article and supplementary materials. Further inquiries can be directed to the corresponding author.

## Abbreviations

ICH: Intracerebral hemorrhage
SAH: Subarachnoid hemorrhage
IVH: Intraventricular hemorrhage
EVs: Extracellular vesicles
SCEVs: Stem cell extracellular vesicles
CAMARADES: Collaborative approach to meta-analysis and review of animal data from experimental stroke
PRISMA: Preferred reporting items for systematic review and meta-analyses
SD: Standard deviation
SE: Standard error
SMD: Standardized mean difference
CI: Confidence interval
MSC: Mesenchymal stem cell
ADSC: Adipose-derived stem cell
mMWM: Modified Morris water maze
mNSS: Modified neurological severity score.

## Additional Points

*Limitation*. The limitations of this meta-analysis are well known. The high heterogeneity of the results may be attributed to the methodological heterogeneity of the studies. Specifically, the types of SCEVs used in the studies, extraction methods, and animal models vary widely. Given the limited number of included studies, a convincing subgroup analysis was not possible. Through random-effects analysis, the risk of misestimation was found to be low. Second, our research only included the available data, and some negative results were less likely to have been published, which could have introduced publication bias. We call for substantial future studies to focus on the effect of SCEVs on ICH. We hope to conduct further studies in the future to validate our findings. Our study is subject to publication bias; however, the results of the splice complementation method suggested that our results were stable. Finally, the animal studies had relatively short follow-up periods. We believe these limitations will be addressed in future studies. This analysis suggests that SCEV therapy may improve neurobehavioral scores after ICH and provides important clues for the design of clinical trials.

## Supplementary Materials

Supplementary 1 Table 1: search strategy for each database. Table 2: excluded articles and reasons for exclusion. Table 3: reviews included potential study and potential study. Table 4: characteristics of the excluded studies about hemorrhagic stroke. Table 5: methodological quality of three studies excluded studies about hemorrhagic stroke.

Supplementary 2 Figure 1: subgroup analysis by animal types in SAH. Figure 2: subgroup analysis by anesthetic drugs in SAH. Figure 3: subgroup analysis by methods of SAH. Figure 4: subgroup analysis by SCEVs types in SAH. Figure 5: subgroup analysis by delivery routes in SAH. Figure 6: subgroup analysis by animal types for the acute neurobehavioral scores in ICH. Figure 7: subgroup analysis by anesthetic drugs for the acute neurobehavioral scores in ICH. Figure 8: subgroup analysis by methods of ICH for the acute neurobehavioral scores. Figure 9: subgroup analysis by SCEVs types for the acute neurobehavioral score in ICH. Figure 10: subgroup analysis by animal types for the subacute neurobehavioral scores in ICH. Figure 11: subgroup analysis by anesthetic drugs for the subacute neurobehavioral scores in ICH. Figure 12: subgroup analysis by methods of ICH for the subacute neurobehavioral scores. Figure 13: subgroup analysis by animal types for the chronic neurobehavioral scores in ICH. Figure 14: subgroup analysis by anesthetic drugs for the chronic neurobehavioral scores in ICH. Figure 15: subgroup analysis by methods of ICH for the chronic neurobehavioral scores. Figure 16: subgroup analysis by SCEVs types for the chronic neurobehavioral scores in ICH.

Supplementary 3 Table 6: PRISMA 2020 checklist.
