# Supplementary material for: Meta-Analysis of the Therapeutic Effects of Stem Cell-Derived Extracellular Vesicles in Rodent Models of Hemorrhagic Stroke
Source: Stem Cells Int. 2024 Jun 27;2024:3390446. doi: 10.1155/2024/3390446 (PMC11390234; doi:10.1155/2024/3390446)
Supplement: Supplementary 1 — Table 1: search strategy for each database. Table 2: excluded articles and reasons for exclusion. Table 3: reviews included potential study and potential study. Table 4: characteristics of the excluded studies about hemorrhagic stroke. Table 5: methodological quality of three studies excluded studies about hemorrhagic stroke. [file 3390446.f1.docx]

Supplement Table S1. Search Strategy for Each Database

| Database | Search strategy |
| --- | --- |
| Pubmed | #1"cerebral hemorrhage"[Mesh Terms] OR "cerebral hemorrhage" [All Fields] |
|  | #2 "hemorrhagic stroke"[Mesh] OR "hemorrhagic stroke"[All Fields] |
|  | #3 "stem cells"[Mesh Terms] OR "stem cells"[All Fields] |
|  | #4"Extracellular vesicles"[MeSH Terms] OR "extracellular vesicles"[All Fields] |
|  | #5 "cell-derived microparticles"[MeSH Terms] OR "cell-derived microparticles"[All Fields] |
|  | #6 "exosomes"[MeSH Terms] OR "exosomes"[All Fields] |
|  | #7 #1 OR #2 |
|  | #8 #4 OR #5 OR #6 |
|  | #9 #7 AND #8 |
|  | #10 #3 AND #9 |
| Embase | #1 'brain hemorrhage'/exp |
|  | #2 'brain hemorrhage' OR 'Cerebrum Hemorrhage' OR 'Cerebrum Hemorrhages' OR 'Cerebral Parenchymal Hemorrhage' OR 'Cerebral Parenchymal Hemorrhages' OR 'Intracerebral Hemorrhage' OR 'Intracerebral Hemorrhages' OR 'Cerebral Hemorrhages' OR 'Cerebral Brain Hemorrhage' OR 'Cerebral Brain Hemorrhages' OR 'Hemorrhagic Strokes' OR 'Subarachnoid Hemorrhagic Stroke' OR 'Subarachnoid Hemorrhagic Strokes' OR 'Intracerebral Hemorrhagic Stroke' OR 'Intracerebral Hemorrhagic Strokes' OR 'Intracerebral Hemorrhage Stroke' OR 'Intracerebral Hemorrhage Strokes' OR |
|  | #3 'stem cell'/exp |
|  | #4 'stem cell' OR 'Stem Cell' OR 'Progenitor Cells' OR 'Progenitor Cell' OR 'Mother Cells' OR 'Mother Cell' OR 'Colony-Forming Unit' OR 'Colony Forming Unit' OR 'Colony-Forming Units' OR 'Colony Forming Units' |
|  | #5 'exosome'/exp |
|  | #6 'membrane microparticle'/exp |
|  | #7 'Extracellular Vesicle' OR 'Exovesicles' OR 'Exovesicle' OR 'Apoptotic Bodies' OR 'Apoptotic Body' OR 'Cell Derived Microparticles' OR 'Cell-Derived Microparticle' OR 'Ectosomes' OR 'Ectosome' OR 'Shedding Microvesicles' OR 'Shedding Microvesicle' OR 'Cell Membrane Microparticles' OR 'Cell Membrane Microparticle' OR 'Circulating Cell-Derived Microparticles' OR 'Circulating Cell Derived Microparticles' OR 'Circulating Cell-Derived Microparticle' |
|  | #8 #1 OR #2 |
|  | #9 #3 OR #4 |
|  | #10 #5 OR #6 OR #7 |
|  | #11 #8 AND #9 AND #10 |
| Web of Science | #1 TS=(cerebral hemorrhage OR Hemorrhage, Cerebrum OR Cerebrum Hemorrhage OR Cerebrum Hemorrhages OR Cerebral Parenchymal Hemorrhage OR Cerebral Parenchymal Hemorrhages OR Hemorrhage, Cerebral Parenchymal OR Hemorrhages, Cerebral Parenchymal OR Parenchymal Hemorrhage, Cerebral OR Parenchymal Hemorrhages, Cerebral OR Intracerebral Hemorrhage OR Hemorrhage, Intracerebral OR Hemorrhages, Intracerebral OR Intracerebral Hemorrhages OR Hemorrhage, Cerebral OR Cerebral Hemorrhages OR Hemorrhages, Cerebral OR Brain Hemorrhage, Cerebral OR Brain Hemorrhages, Cerebral OR Cerebral Brain Hemorrhage OR Cerebral Brain Hemorrhages OR Hemorrhage, Cerebral Brain OR Hemorrhages, Cerebral Brain) |
|  | #2 TS=(hemorrhagic stroke OR Hemorrhagic Strokes OR Stroke, Hemorrhagic OR Subarachnoid Hemorrhagic Stroke OR Hemorrhagic Stroke, Subarachnoid OR Stroke, Subarachnoid Hemorrhagic OR Subarachnoid Hemorrhagic Strokes OR Intracerebral Hemorrhagic Stroke OR Hemorrhagic Stroke, Intracerebral OR Intracerebral Hemorrhagic Strokes OR Stroke, Intracerebral Hemorrhagic OR Intracerebral Hemorrhage Stroke OR Hemorrhage Stroke, Intracerebral OR Intracerebral Hemorrhage Strokes OR Stroke, Intracerebral Hemorrhage) |
|  | #3 TS=(stem cells OR Cell, Stem OR Cells, Stem OR Stem Cell OR Progenitor Cells OR Cell, Progenitor OR Cells, Progenitor OR Progenitor Cell OR Mother Cells OR Cell, Mother OR Cells, Mother OR Mother Cell OR Colony-Forming Unit OR Colony Forming Unit OR Colony-Forming Units OR Colony Forming Units) |
|  | #4 TS=(Extracellular vesicles OR Extracellular Vesicle OR Vesicle, Extracellular OR Vesicles, Extracellular OR Exovesicles OR Exovesicle OR Apoptotic Bodies OR Apoptotic Body OR Bodies, Apoptotic OR Body, Apoptotic) |
|  | #5 TS=(Cell-Derived Microparticles OR Cell Derived Microparticles OR Cell-Derived Microparticle OR Microparticle, Cell-Derived OR Ectosomes OR Ectosome OR Microparticles, Cell-Derived OR Microparticles, Cell Derived OR Shedding Microvesicles OR Microvesicle, Shedding OR Microvesicles, Shedding OR Shedding Microvesicle OR Cell Membrane Microparticles OR Cell Membrane Microparticle OR Membrane Microparticle, Cell OR Membrane Microparticles, Cell OR Microparticle, Cell Membrane OR Microparticles, Cell Membrane OR Circulating Cell-Derived Microparticles OR Cell-Derived Microparticle, Circulating OR Cell-Derived Microparticles, Circulating OR Circulating Cell Derived Microparticles OR Circulating Cell-Derived Microparticle OR Microparticle, Circulating Cell-Derived OR Microparticles, Circulating Cell-Derived) |
|  | #6 TS=(exosomes) |
|  | #7 #1 OR #2 |
|  | #8 #4 OR #5 OR #6 |
|  | #9 #3 AND #7 AND #8 |

Supplement Table S2. Excluded Articles and Reasons for Exclusion

| Excluded Articles | Reasons for Exclusion |
| --- | --- |
| Stiver 2004[1] | The ICH model was not used in the article. |
| Van Ierssel 2014[2] | The ICH model and SCs were not used in the article. |
| Chen 2015[3] | Review about SAH. |
| Van Ierssel 2015[4] | Animal experiments were not used in the article. |
| Huang 2016[5] | The ICH model and SCs were not used in the article. |
| Quartini 2016[6] | The exosomes/EVs were not used in the article. |
| Ortega 2016[7] | Conference abstract. |
| Wang 2016[8] | The ICH model was not used in the article. |
| Zhang 2016[9] | The exosomes/EVs were not used in the article. |
| Xin 2017[10] | Conference abstract. |
| Lapchak 2018[11] | The model used in the article is the small-clot embolized rabbits. |
| Min 2018[12] | Exosomes/EVs from stem cells were not used in in vivo experiments. |
| Otero-Ortega 2018[13] | Article has been retracted in July,2021. |
| Shen 2018[14] | The outcome did not include the neurobehavioral score. |
| Venkat 2018[15] | Review about exosome therapy in diabetic stroke. |
| Vogel 2018[16] | Review about neural plasticity and neuroregeneration,not involved ICH. |
| Webb 2018[17] | The model used in the article is the porcine model of ischemic stroke. |
| Zhang 2018[18] | Exosomes/EVs from stem cells were not used in in vivo experiments. |
| Boruczkowski 2019[19] | Review about autologous cord blood. |
| Chen 2019[20] | Article is about stem cell therapy for cerebral hemorrhage, but does not involve exosomes/EVs. |
| Vaes 2019[21] | Review about white matter injury. |
| Wang 2019[22] | Conference abstract. |
| Zhu 2019[23] | Review about ischemic stroke. |
| Ali 2020[24] | This paper mainly focused on ischemia reperfusion injury, but did not focus on ICH. |
| Chung 2020[25] | Conference abstract. |
| Duan 2020[26] | The neurofunctional score in the article is the median, not the mean±SD/SE. |
| El Moshy 2020[27] | Review about the application of dental MSC secretome/conditioned medium. |
| Gao 2020[28] | Article focused on cerebral hemorrhage, but does not involve stem cell exosomes or EVs. |
| Gao 2020[29] | Review about ischemic stroke. |
| Lengel 2020[30] | Review about stem cell therapy for pediatric traumatic brain injury. |
| Li 2020[31] | There are no in vivo experiments in the article |
| Mello 2020[32] | Article used the intravenous injection of human umbilical cord mesenchymal cells in the model of moderate to severe intracerebral hemorrhage, rather than exosomes or EVs. |
| Williams 2020[33] | The animal model studied was traumatic brain injury, not ICH.And the animal model is not a rodent, but a porcine. |
| Zhang 2020[34] | Review about ischemic stroke. |
| Alwjwaj 2021[35] | Review about ischemic stroke. |
| Amari 2021[36] | Review about the nature and roles of mitochondrial extracellular vesicles. |
| Baumgartner 2021[37] | Review about progenitor cell therapy for traumatic brain injury. |
| Bobinger 2021[38] | Animal experiments were not used in the article. |
| Boltze 2021[39] | Review about cerebrovascular diseases. |
| Caicedo 2021[40] | Review about extracellular mitochondria in the cerebrospinal fluid. |
| Dekker 2021[41] | The animal model used in the experiment was pigs,not rodents. |
| Di Cesare Mannelli 2021[42] | Editorial. |
| Garbuzova-Davis 2021[43] | Article focused on stem cell-derived extracellular vesicles in amyotrophic lateral sclerosis. |
| Haupt 2021[44] | Mesenchymal stem cells were preconditioned with Lithium.Article focused on middle cerebral artery occlusion (MCAO) model, not ICH model. |
| Hurd 2021[45] | Review about ischemic stroke. |
| Liu 2021[46] | The exosomes/EVs were not used in the article. |
| Liu 2021[47] | Review about intercellular mitochondrial. |
| Liu 2021[48] | The neurofunctional score in the article is the median, not the mean±SD/SE. |
| Saghazadeh 2021[49] | The exosomes/EVs were not used in the article. |
| Signorelli 2021[50] | Article focused on parkinson’s disease. |
| Tang 2021[51] | The exosomes/EVs were not used in the article. |
| Zanirati 2021[52] | Review about stem cell-based therapy for COVID-19 and ARDS. |
| Bambakidis 2022[53] | The animal model studied was traumatic brain injury, not ICH.And the animal model is not a rodent, but a porcine. |
| Chang 2022[54] | The article is not about ICH. |
| Chaubey 2022[55] | Review about stem cells in neonatal diseases. |
| Delavogia 2022[56] | Review focused on neonatal diseases. |
| Ejma 2022[57] | Review about stem cells in the therapy of stroke. |
| Huang 2022[58] | Review about hemorrhagic shock |
| Huang 2022[59] | Article focused on ischemic stroke, not ICH model. |
| He 2022[60] | Article focused on SAH model, not ICH model. |
| Kim 2022[61] | Conference abstract. |
| Liu 2022[62] | Review about exosomes in the treatment of Parkinson's disease. |
| Monsour 2022[63] | Review about epigenetic modifications in cell-based therapies for stroke. |
| Pardridge 2022[64] | Review about brain drug delivery. |
| Qiu 2022[65] | Article focused on ischemic stroke, not ICH model. |
| Shah 2022[66] | The exosomes/EVs were not used in the article. |
| Thomas 2022[67] | Article focuses on in vitro experiments and does not involve animal models. |
| Tian 2022[68] | The article focuses on ischemic stroke, not cerebral hemorrhage. |
| Venkat 2022[69] | Article focused on ischemic stroke in T2DM rats, not ICH model. |
| Wang 2022[70] | Review about traumatic brain injury. |
| Wang 2022[71] | Chinese article. |
| Wang 2022[72] | Article focused on tumor brain metastases. |
| Yang 2022[73] | Review about mesenchymal stem cell application and its therapeutic mechanisms in ICH. |
| Douceau 2023[74] | Review about neural development. |
| Fasoulakis 2023[75] | Review focused on inflammatory factors and did not involve cerebral hemorrhage. |
| Tung 2023[76] | Review about stem cell secretome in neonatal diseases. |

**References**

1. Stiver SI, Tan X, Brown LF, Hedley-Whyte ET, Dvorak HF. VEGF-A angiogenesis induces a stable neovasculature in adult murine brain. JOURNAL OF NEUROPATHOLOGY AND EXPERIMENTAL NEUROLOGY. 2004;63(8):841-55.

2. Van Ierssel SH, Jorens PG, Van Craenenbroeck EM, Conraads VM. The endothelium, a protagonist in the pathophysiology of critical illness: Focus on cellular markers. BioMed Research InternationalBioMed Res. Int.. 2014;2014:-.

3. Chen Y, Li Q, Tang J, Feng H, Zhang JH. The evolving roles of pericyte in early brain injury after subarachnoid hemorrhage. Brain ResearchBrain Res.. 2015;1623:110-22.

4. van Ierssel SH, Conraads VM, Van Craenenbroeck EM, Liu Y, Maas AIR, Parizel PM, Hoymans VY, Vrints CJ, Jorens PG. Endothelial dysfunction in acute brain injury and the development of cerebral ischemia. Journal of Neuroscience ResearchJ. Neurosci. Res.. 2015;93(6):866-72.

5. Huang H, Zhang Q, Liu JJ, Hao HJ, Jiang CG, Han WD. Granulocyte-Colony Stimulating Factor (G-CSF) Accelerates Wound Healing in Hemorrhagic Shock Rats by Enhancing Angiogenesis and Attenuating Apoptosis. MEDICAL SCIENCE MONITOR. 2017;23:2644-53.

6. Quartini A, Iannitelli A, Bersani G. Lithium: from mood stabilizer to putative cognitive enhancer. Neural Regeneration Research. 2016;11(8):1234-5.

7. Ortega LO, Fernández MG, Frutos BR, Cejudo JR, Llorente IL, Fuentes B, Medina E, Carmichael ST, Tejedor ED. Functional recovery and white matter repair after exosomes administration in different experimental animal models of stroke. StrokeStroke. 2016;47:-.

8. Wang GH, Liu Y, Wu XB, Lu Y, Liu J, Qin YR, Li T, Duan HF. Neuroprotective effects of human umbilical cord-derived mesenchymal stromal cells combined with nimodipine against radiation-induced brain injury through inhibition of apoptosis. Cytotherapy. 2016;18(1):53-64.

9. Zhang N, Luo Y, He L, Zhou L, Wu W. A self-assembly peptide nanofibrous scaffold reduces inflammatory response and promotes functional recovery in a mouse model of intracerebral hemorrhage. Nanomedicine-Nanotechnology Biology and Medicine. 2016;12(5):1205-17.

10. Xin H, Wang F, Li Y, Lu Q, Cheung WL, Zhang Y, Zhang ZG, Choppt M. Secondary Release of Exosomes From Astrocytes Contributes to the Increase in Neural Plasticity and Improvement of Functional Recovery After Stroke in Rats Treated With Exosomes Harvested From MicroRNA 133b-Overexpressing Multipotent Mesenchymal Stromal Cells. CELL TRANSPLANTATION. 2017;26(2):243-57.

11. Lapchak PA, Boitano PD, de Couto G, Marbán E. Intravenous xenogeneic human cardiosphere-derived cell extracellular vesicles (exosomes) improves behavioral function in small-clot embolized rabbits. Experimental NeurologyExp. Neurol.. 2018;307:109-17.

12. Min S, Kim OJ, Bae J, Chung TN. Effect of pretreatment with the NADPH oxidase inhibitor apocynin on the therapeutic efficacy of human placenta-derived mesenchymal stem cells in intracerebral hemorrhage. International Journal of Molecular SciencesInt. J. Mol. Sci.. 2018;19(11):-.

13. Otero-Ortega L, Gómez de Frutos MC, Laso-García F, Rodríguez-Frutos B, Medina-Gutiérrez E, López JA, Vázquez J, Díez-Tejedor E, Gutiérrez-Fernández M. Exosomes promote restoration after an experimental animal model of intracerebral hemorrhage. J Cereb Blood Flow Metab. 2018;38(5):767-79.

14. Shen H, Yao X, Li H, Li X, Zhang T, Sun Q, Ji C, Chen G. Role of Exosomes Derived from miR-133b Modified MSCs in an Experimental Rat Model of Intracerebral Hemorrhage. J Mol Neurosci. 2018;64(3):421-30.

15. Venkat P, Chopp M, Chen J. Cell-Based and Exosome Therapy in Diabetic Stroke. Stem Cells Translational MedicineStem Cells Transl. Med.. 2018;7(6):451-5.

16. Vogel A, Upadhya R, Shetty AK. Neural stem cell derived extracellular vesicles: Attributes and prospects for treating neurodegenerative disorders. EBioMedicineEBioMedicine. 2018;38:273-82.

17. Webb R, Kaiser EE, Jurgielewicz BJ, Spellicy S, Scoville S, Thompson T, Swetenburg RL, West F, Stice S. Human neural stem cell extracellular vesicles improve recovery in a porcine model of ischemic stroke. Journal of Extracellular VesiclesJ. Extracell. Vesicles. 2018;7:105-105.

18. Zhang H, Wang Y, Lv Q, Gao J, Hu L, He Z. MicroRNA-21 overexpression promotes the neuroprotective efficacy of mesenchymal stem cells for treatment of intracerebral hemorrhage. Frontiers in NeurologyFront. Neurol.. 2018;9(NOV):-.

19. Boruczkowski D, Pujal J-M, Zdolińska-Malinowska I. Autologous cord blood in children with cerebral palsy: A review. International Journal of Molecular SciencesInt. J. Mol. Sci.. 2019;20(10):-.

20. Chen KH, Lin KC, Wallace CG, Li YC, Shao PL, Chiang JY, Sung PH, Yip HK. Human induced pluripotent stem cell-derived mesenchymal stem cell therapy effectively reduced brain infarct volume and preserved neurological function in rat after acute intracranial hemorrhage. Am J Transl Res. 2019;11(9):6232-48.

21. Vaes JEG, Vink MA, De Theije CGM, Hoebeek FE, Benders MJNL, Nijboer CHA. The potential of stem cell therapy to repair white matter injury in preterm infants: Lessons learned from experimental models. Frontiers in PhysiologyFront. Physiol.. 2019;10(MAY):-.

22. Wang J, Pan Q, Zhao B, Ma X, Bihl JC. Overexpression of ACE2 boosts the therapeutic effects of endothelial progenitor cells derived exosomes on hemorrhagic stroke. HypertensionHypertension. 2019;74:-.

23. Zhu Z, Zheng L, Li Y, Huang T, Chao Y-C, Pan L, Zhu H, Zhao Y, Yu W, Li P. Potential immunotherapeutic targets on myeloid cells for neurovascular repair after ischemic stroke. Frontiers in GeneticsFront. Genet.. 2019;10(JUL):-.

24. Ali M, Pham A, Wang X, Wolfram J, Pham S. Extracellular vesicles for treatment of solid organ ischemia-reperfusion injury. AMERICAN JOURNAL OF TRANSPLANTATION. 2020;20(12):3294-307.

25. Chung TN, Jo J. Effect of mesenchymal stem cell-derived exosomes on the intracerebral hemorrhage in acute stage. International Journal of StrokeInt. J. Stroke. 2020;15(1 SUPPL):148-148.

26. Duan S, Wang F, Cao J, Wang C. Exosomes Derived from MicroRNA-146a-5p-Enriched Bone Marrow Mesenchymal Stem Cells Alleviate Intracerebral Hemorrhage by Inhibiting Neuronal Apoptosis and Microglial M1 Polarization. Drug Des Devel Ther. 2020;14:3143-58.

27. El Moshy S, Radwan IA, Rady D, Abbass MMS, El-Rashidy AA, Sadek KM, Dörfer CE, Fawzy El-Sayed KM. Dental Stem Cell-Derived Secretome/Conditioned Medium: The Future for Regenerative Therapeutic Applications. Stem Cells InternationalStem Cells Intl.. 2020;2020:-.

28. Gao L, Li P-P, Shao T-Y, Mao X, Qi H, Wu B-S, Shan M, Ye L, Cheng H-W. Neurotoxic role of interleukin-17 in neural stem cell differentiation after intracerebral hemorrhage. Neural Regeneration ResearchNeural. Regen. Res.. 2020;15(7):1350-9.

29. Gao L, Song Z, Mi J, Hou P, Xie C, Shi J, Li Y, Manaenko A. The effects and underlying mechanisms of cell therapy on blood-brain barrier integrity after ischemic stroke. Current NeuropharmacologyCurr. Neuropharmacol.. 2020;18(12):1213-26.

30. Lengel D, Sevilla C, Romm ZL, Huh JW, Raghupathi R. Stem Cell Therapy for Pediatric Traumatic Brain Injury. Frontiers in NeurologyFront. Neurol.. 2020;11:-.

31. Li YC, Wang JJ, Chen SZ, Wu P, Xu SC, Wang CL, Shi HZ, Bihl J. miR-137 boosts the neuroprotective effect of endothelial progenitor cell-derived exosomes in oxyhemoglobin-treated SH-SY5Y cells partially via COX2/PGE2 pathway. Stem Cell Res Ther. 2020;11(1):-.

32. Mello TG, Rosado-de-Castro PH, Pereira Campos RM, Vasques JF, Rangel-Junior WS, Rezende de Mattos RSdA, Puig-Pijuan T, Foerster BU, Gutfilen B, Lopes Souza SA, et al. Intravenous Human Umbilical Cord-Derived Mesenchymal Stromal Cell Administration in Models of Moderate and Severe Intracerebral Hemorrhage. STEM CELLS AND DEVELOPMENT. 2020;29(9):586-98.

33. Williams AM, Higgins GA, Bhatti UF, Biesterveld BE, Dekker SE, Kathawate RG, Tian YZ, Wu ZY, Kemp MT, Wakam GK, et al. Early treatment with exosomes following traumatic brain injury and hemorrhagic shock in a swine model promotes transcriptional changes associated with neuroprotection. Journal of Trauma and Acute Care Surgery. 2020;89(3):536-43.

34. Zhang S, Lachance BB, Moiz B, Jia X. Optimizing stem cell therapy after ischemic brain injury. Journal of StrokeJ. Stroke. 2020;22(3):286-305.

35. Alwjwaj M, Kadir R, Bayraktutan U. The secretome of endothelial progenitor cells: a potential therapeutic strategy for ischemic stroke. Neural Regeneration Research. 2021;16(8):1483-9.

36. Amari L, Germain M. Mitochondrial Extracellular Vesicles – Origins and Roles. Frontiers in Molecular NeuroscienceFront. Mol. Neurosci.. 2021;14:-.

37. Baumgartner JE, Baumgartner LS, Baumgartner ME, Moore EJ, Messina SA, Seidman MD, Shook DR. Progenitor cell therapy for acquired pediatric nervous system injury: Traumatic brain injury and acquired sensorineural hearing loss. Stem Cells Translational MedicineStem Cells Transl. Med.. 2021;10(2):164-80.

38. Bobinger T, Roeder SS, Spruegel MI, Froehlich K, Beuscher VD, Hoelter P, Lücking H, Corbeil D, Huttner HB. Variation of membrane particle–bound CD133 in cerebrospinal fluid of patients with subarachnoid and intracerebral hemorrhage. Journal of NeurosurgeryJ. Neurosurg.. 2021;134(2):600-7.

39. Boltze J, Aronowski JA, Badaut J, Buckwalter MS, Caleo M, Chopp M, Dave KR, Didwischus N, Dijkhuizen RM, Doeppner TR, et al. New Mechanistic Insights, Novel Treatment Paradigms, and Clinical Progress in Cerebrovascular Diseases. Frontiers in Aging NeuroscienceFront. Aging Neurosci.. 2021;13:-.

40. Caicedo A, Zambrano K, Sanon S, Gavilanes AWD. Extracellular mitochondria in the cerebrospinal fluid (CSF): Potential types and key roles in central nervous system (CNS) physiology and pathogenesis. MitochondrionMitochondrion. 2021;58:255-69.

41. Dekker SE, Bambakidis T, Williams AM, Biesterveld B, Bhatti U, Li Y, Pickell Z, Buller B, Alam HB. Early Transfusion with Mesenchymal Stem Cell Derived Extracellular Vesicles: A New Transfusion Strategy for Life-Threatening Hemorrhage and Traumatic Brain Injury. BLOOD. 2021;138.

42. Di Cesare Mannelli L, Ceruti S, Orellana JA. Editorial: Astrocytes, a Kaleidoscope of Diversities, a Pharmacological Horizon. Frontiers in PharmacologyFront. Pharmacol.. 2021;12:-.

43. Garbuzova-Davis S, Borlongan CV. Stem cell-derived extracellular vesicles as potential mechanism for repair of microvascular damage within and outside of the central nervous system in amyotrophic lateral sclerosis: Perspective schema. Neural Regeneration ResearchNeural. Regen. Res.. 2021;16(4):680-1.

44. Haupt M, Zheng X, Kuang YY, Lieschke S, Janssen L, Bosche B, Jin FY, Hein K, Kilic E, Venkataramani V, et al. Lithium modulates miR-1906 levels of mesenchymal stem cell-derived extracellular vesicles contributing to poststroke neuroprotection by toll-like receptor 4 regulation. Stem Cells Translational Medicine. 2021;10(3):357-73.

45. Hurd MD, Goel I, Sakai Y, Teramura Y. Current status of ischemic stroke treatment: From thrombolysis to potential regenerative medicine. Regenerative TherapyRegen. Ther.. 2021;18:408-17.

46. Liu JY, He JL, Huang Y, Ge LT, Xiao H, Zeng LW, Jiang Z, Lu M, Hu ZP. Hypoxia-preconditioned mesenchymal stem cells attenuate microglial pyroptosis after intracerebral hemorrhage. Ann Transl Med. 2021;9(17):-.

47. Liu D, Gao Y, Liu J, Huang Y, Yin J, Feng Y, Shi L, Meloni BP, Zhang C, Zheng M, et al. Intercellular mitochondrial transfer as a means of tissue revitalization. Signal Transduction and Targeted TherapySignal Transduct. Target. Ther.. 2021;6(1):-.

48. Liu Z, Wang B, Guo Q. MiR-26b-5p-modified hUB-MSCs derived exosomes attenuate early brain injury during subarachnoid hemorrhage via MAT2A-mediated the p38 MAPK/STAT3 signaling pathway. Brain Research BulletinBrain Res. Bull.. 2021;175:107-15.

49. Saghazadeh A, Rezaei N. Biosensing surfaces and therapeutic biomaterials for the central nervous system in COVID-19. Emergent Materials. 2021;4(1):293-312.

50. Signorelli P, Conte C, Albi E. The multiple roles of sphingomyelin in parkinson’s disease. BiomoleculesBiomolecules. 2021;11(9):-.

51. Tang B, Song M, Xie X, Le D, Tu Q, Wu X, Chen M. Tumor Necrosis Factor-stimulated Gene-6 (TSG-6) Secreted by BMSCs Regulates Activated Astrocytes by Inhibiting NF-κB Signaling Pathway to Ameliorate Blood Brain Barrier Damage After Intracerebral Hemorrhage. Neurochem Res. 2021;46(9):2387-402.

52. Zanirati G, Provenzi L, Libermann LL, Bizotto SC, Ghilardi IM, Marinowic DR, Shetty AK, Da Costa JC. Stem cell-based therapy for COVID-19 and ARDS: a systematic review. npj Regenerative Medicinenpj Regen. Med.. 2021;6(1):-.

53. Bambakidis T, Dekker SE, Williams AM, Biesterveld B, Bhatti UF, Liu BL, Li YQ, Pickell Z, Buller B, Alam HB. Early Treatment With a Single Dose of Mesenchymal Stem Cell Derived Extracellular Vesicles Modulates the Brain Transcriptome to Create Neuroprotective Changes in a Porcine Model of Traumatic Brain Injury and Hemorrhagic Shock. SHOCK. 2022;57(2):281-90.

54. Chang YS. Extracellular vesicles derived from mesenchymal stem cells for the treatment of intractable neonatal diseases. Tissue Engineering Part A. 2022;28:27-27.

55. Chaubey S, Bhandari V. Stem cells in neonatal diseases: An overview. Seminars in Fetal and Neonatal MedicineSemin. Fetal Neonatal Med.. 2022;27(1).

56. Delavogia E, Ntentakis DP, Cortinas JA, Fernandez-Gonzalez A, Mitsialis SA, Kourembanas S. Mesenchymal Stromal/Stem Cell Extracellular Vesicles and Perinatal Injury: One Formula for Many Diseases. STEM CELLS. 2022;40(11):991-1007.

57. Ejma M, Madetko N, Brzecka A, Alster P, Budrewicz S, Koszewicz M, Misiuk-Hojło M, Tomilova IK, Somasundaram SG, Kirkland CE. The Role of Stem Cells in the Therapy of Stroke. Current NeuropharmacologyCurr. Neuropharmacol.. 2022;20(3):630-47.

58. Huang Q, Gao S, Yao Y, Wang Y, Li J, Chen J, guo C, Zhao D, Li X. Innate immunity and immunotherapy for hemorrhagic shock. Frontiers in ImmunologyFront. Immunol.. 2022;13.

59. Huang L, Hua L, Zhang X. The Exosomal MicroRNA Profile Is Responsible for the Mesenchymal Stromal Cell Transplantation-Induced Improvement of Functional Recovery after Stroke in Rats. NeuroImmunoModulationNeuroImmunomodulation. 2022;29(2):151-60.

60. He J, Liu J, Huang Y, Lan Z, Tang X, Hu Z. Mesenchymal stem cells-derived therapies for subarachnoid hemorrhage in preclinical rodent models: a meta-analysis. Stem Cell Research and TherapyStem Cell Res. Ther.. 2022;13(1):-.

61. Kim HY. Magnet-assisted systemic delivery of artificial extracellular vesicles to injured central nervous system. Tissue Engineering Part A. 2022;28:28-28.

62. Liu S-F, Li L-Y, Zhuang J-L, Li M-M, Ye L-C, Chen X-R, Lin S, Chen C-N. Update on the application of mesenchymal stem cell-derived exosomes in the treatment of Parkinson's disease: A systematic review. Frontiers in NeurologyFront. Neurol.. 2022;13.

63. Monsour M, Gordon J, Lockard G, Alayli A, Elsayed B, Connolly J, Borlongan CV. Minor Changes for a Major Impact: A Review of Epigenetic Modifications in Cell-Based Therapies for Stroke. INTERNATIONAL JOURNAL OF MOLECULAR SCIENCES. 2022;23(21).

64. Pardridge WM. A historical review of brain drug delivery. PharmaceuticsPharmaceutics. 2022;14(6).

65. Qiu L, Cai Y, Geng Y, Yao X, Wang L, Cao H, Zhang X, Wu Q, Kong D, Ding D, et al. Mesenchymal stem cell-derived extracellular vesicles attenuate tPA-induced blood–brain barrier disruption in murine ischemic stroke models. Acta BiomaterialiaActa Biomater.. 2022;154:424-42.

66. Shah JS, Macaitis J, Lundquist B, Johnstone B, Coleman M, Jefferson MA, Glaser J, Rodriguez AR, Cardin S, Wang H-C, et al. Evaluating Thera-101 as a Low-Volume Resuscitation Fluid in a Model of Polytrauma. International Journal of Molecular SciencesInt. J. Mol. Sci.. 2022;23(20).

67. Thomas JJ, Harp KO, Bashi A, Hood JL, Botchway F, Wilson MD, Thompson WE, Stiles JK, Driss A. MiR-451a and let-7i-5p loaded extracellular vesicles attenuate heme-induced inflammation in hiPSC-derived endothelial cells. Front Immunol. 2022;13:1082414.

68. Tian J, Yao H, Liu Y, Wang X, Wu J, Wang J, Yu D, Xie Y, Gao J, Zhu Y, et al. Extracellular vesicles from bone marrow stromal cells reduce the impact of stroke on glial cell activation and blood brain-barrier permeability via a putative miR-124/PRX1 signalling pathway. EUROPEAN JOURNAL OF NEUROSCIENCE. 2022;56(2):3786-805.

69. Venkat P, Chopp M. Exosome treatment for stroke with diabetic comorbidity. Neural Regeneration ResearchNeural. Regen. Res.. 2022;17(2):315-7.

70. Wang J-P, Li C, Ding W-C, Peng G, Xiao G-L, Chen R, Cheng Q. Research Progress on the Inflammatory Effects of Long Non-coding RNA in Traumatic Brain Injury. Frontiers in Molecular NeuroscienceFront. Mol. Neurosci.. 2022;15:-.

71. Wang X, Liu Y, Xu J, Long Q, Wang T, Zhong J. Neuroprotective effect of umbilical cord mesenchymal stem cell-derived exosomes on hippocampal neurons in mice with intracerebral hemorrhage. Chinese Journal of Tissue Engineering ResearchChin. J. Tissue Eng. Res.. 2022;26(31):4928-34.

72. Wang Y, Chen R, Wa Y, Ding S, Yang Y, Liao J, Tong L, Xiao G. Tumor Immune Microenvironment and Immunotherapy in Brain Metastasis From Non-Small Cell Lung Cancer. Frontiers in ImmunologyFront. Immunol.. 2022;13:-.

73. Yang G, Fan X, Mazhar M, Yang S, Xu H, Dechsupa N, Wang L. Mesenchymal Stem Cell Application and Its Therapeutic Mechanisms in Intracerebral Hemorrhage. Frontiers in Cellular NeuroscienceFront. Cell. Neurosci.. 2022;16.

74. Douceau S, Deutsch Guerrero T, Ferent J. Establishing Hedgehog Gradients during Neural Development. CellsCells. 2023;12(2).

75. Fasoulakis Z, Koutras A, Ntounis T, Antsaklis P, Theodora M, Valsamaki A, Daskalakis G, Kontomanolis EN. Inflammatory Molecules Responsible for Length Shortening and Preterm Birth. Cells. 2023;12(2).

76. Tung S, Delavogia E, Fernandez-Gonzalez A, Mitsialis SA, Kourembanas S. Harnessing the therapeutic potential of the stem cell secretome in neonatal diseases. Seminars in PerinatologySemin. Perinatol.. 2023.

Supplement Table S3. Reviews included potential study and potential study

| Reviews | Related articles |
| --- | --- |
| Rosado-de-Castro 2016[1] | - |
| Bedini 2018[2] | Otero-Ortega 2018[3]* |
| Dabrowska 2019[4] | - |
| Shi 2019[5] | - |
| Singh 2020[6] | - |
| Azizi 2020[7] | - |
| Cai 2020[8] | Han 2018[9], Shen 2018[10] |
| Nasirishargh 2021[11] | Shen 2018[10], Duan 2020[12] |
| Nakano 2021[13] | Otero-Ortega 2018[3]* |
| Cetin 2021[14] | Shen 2018[10] |
| Chuang 2022[15] |  |
| Lee 2022[16] |  |
| Zhang 2022[17] |  |
| Zhang 2022[18] | - |
| Zhou 2022[19] |  |
| Hirsch 2023[20] |  |
| Jafarinia 2023[21] |  |
| Zou 2023[22] |  |

*Otero-Ortega 2018[3] has been retracted in July,2021.

**References**

1. Rosado-de-Castro PH, de Carvalho FG, de Freitas GR, Mendez-Otero R, Pimentel-Coelho PM. Review of Preclinical and Clinical Studies of Bone Marrow-Derived Cell Therapies for Intracerebral Hemorrhage. Stem Cells Int. 2016;2016:-.

2. Bedini G, Bersano A, Zanier ER, Pischiutta F, Parati EA. Mesenchymal Stem Cell Therapy in Intracerebral Haemorrhagic Stroke. Curr Med Chem. 2018;25(19):2176-97.

3. Otero-Ortega L, Gómez de Frutos MC, Laso-García F, Rodríguez-Frutos B, Medina-Gutiérrez E, López JA, Vázquez J, Díez-Tejedor E, Gutiérrez-Fernández M. Exosomes promote restoration after an experimental animal model of intracerebral hemorrhage. J Cereb Blood Flow Metab. 2018;38(5):767-79.

4. Dabrowska S, Andrzejewska A, Lukomska B, Janowski M. Neuroinflammation as a target for treatment of stroke using mesenchymal stem cells and extracellular vesicles. J Neuroinflammation. 2019;16(1):-.

5. Shi B, Yang H, Manaenko A, Lu JF, Mei QY, Hu Q. Potential of Exosomes for the Treatment of Stroke. Cell Transplant. 2019;28(6):662-70.

6. Singh M, Pandey PK, Bhasin A, Padma MV, Mohanty S. Application of Stem Cells in Stroke: A Multifactorial Approach. Front Neurosci. 2020;14:-.

7. Azizi F, Askari S, Javadpour P, Hadjighassem M, Ghasemi R. Potential role of exosome in post-stroke reorganization and/or neurodegeneration. EXCLI JournalEXCLI J.. 2020;19:1590-606.

8. Cai Y, Liu W, Lian L, Xu Y, Bai X, Xu S, Zhang J. Stroke treatment: Is exosome therapy superior to stem cell therapy. BiochimieBiochimie. 2020;179:190-204.

9. Han Y, Seyfried D, Meng Y, Yang D, Schultz L, Chopp M, Seyfried D. Multipotent mesenchymal stromal cell-derived exosomes improve functional recovery after experimental intracerebral hemorrhage in the rat. J Neurosurg. 2018;131(1):290-300.

10. Shen H, Yao X, Li H, Li X, Zhang T, Sun Q, Ji C, Chen G. Role of Exosomes Derived from miR-133b Modified MSCs in an Experimental Rat Model of Intracerebral Hemorrhage. J Mol Neurosci. 2018;64(3):421-30.

11. Nasirishargh A, Kumar P, Ramasubramanian L, Clark K, Hao DK, Lazar SV, Wang AJ. Exosomal microRNAs from mesenchymal stem/stromal cells: Biology and applications in neuroprotection. World J Stem Cells. 2021;13(7):776-94.

12. Duan S, Wang F, Cao J, Wang C. Exosomes Derived from MicroRNA-146a-5p-Enriched Bone Marrow Mesenchymal Stem Cells Alleviate Intracerebral Hemorrhage by Inhibiting Neuronal Apoptosis and Microglial M1 Polarization. Drug Des Devel Ther. 2020;14:3143-58.

13. Nakano M, Fujimiya M. Potential effects of mesenchymal stem cell derived extracellular vesicles and exosomal miRNAs in neurological disorders. Neural Regeneration ResearchNeural. Regen. Res.. 2021;16(12):2359-66.

14. Cetin Z, Saygili EI, Görgisen G, Sokullu E. Preclinical Experimental Applications of miRNA Loaded BMSC Extracellular Vesicles. Stem Cell Reviews and ReportsStem Cell Rev. Rep.. 2021;17(2):471-501.

15. Chuang L, Longwang T, Heshan Z, Chi Z. Adipose-derived mesenchymal stem cell exosomes for treating traumatic central nervous system injury. Chinese Journal of Tissue Engineering ResearchChin. J. Tissue Eng. Res.. 2023;27(19):3061-9.

16. Lee EC, Ha TW, Lee DH, Hong DY, Park SW, Lee JY, Lee MR, Oh JS. Utility of Exosomes in Ischemic and Hemorrhagic Stroke Diagnosis and Treatment. Int J Mol Sci. 2022;23(15).

17. Zhang L, Mao L, Wang H. The Neuroprotection Effects of Exosome in Central Nervous System Injuries: a New Target for Therapeutic Intervention. Molecular NeurobiologyMol. Neurobiol.. 2022;59(12):7152-69.

18. Zhang YC, Dong NJ, Hong HL, Qi JX, Zhang SB, Wang J. Mesenchymal Stem Cells: Therapeutic Mechanisms for Stroke. Int J Mol Sci. 2022;23(5):-.

19. Zhou JF, Xiong Y, Kang X, Pan Z, Zhu Q, Goldbrunner R, Stavrinou L, Lin S, Hu W, Zheng F, et al. Application of stem cells and exosomes in the treatment of intracerebral hemorrhage: an update. Stem Cell Res Ther. 2022;13(1):281.

20. Hirsch Y, Geraghty JR, Reiter CR, Katz EA, Little CF, Tobin MK, Testai FD. Unpacking the Role of Extracellular Vesicles in Ischemic and Hemorrhagic Stroke: Pathophysiology and Therapeutic Implications. Transl Stroke Res. 2023;14(2):146-59.

21. Jafarinia M, Farrokhi MR, Ganjalikhani Hakemi M, Cho WC. The role of miRNAs from mesenchymal stem/stromal cells-derived extracellular vesicles in neurological disorders. Human CellHum. Cell. 2023;36(1):62-75.

22. Zou Y, Liao L, Dai J, Mazhar M, Yang G, Wang H, Dechsupa N, Wang L. Mesenchymal stem cell-derived extracellular vesicles/exosome: A promising therapeutic strategy for intracerebral hemorrhage. Regenerative TherapyRegen. Ther.. 2023;22:181-90.

Supplement Table S4.Characteristics of the excluded studies about hemorrhagic stroke.

| Author(Year) | Country | Types | Species (Gender) | Weight/Year | Anesthetic | Molding method | No. of Treated/Controls Animals | SC Species | EVs Dose | SC route | Time of administration | Assessment time | Neurobehavioral score |
| --- | --- | --- | --- | --- | --- | --- | --- | --- | --- | --- | --- | --- | --- |
| Shen 2018[1] | China | ICH | SD rats(male) | 300-350g | 4% chloral hydrate (10 ml/kg, i.p.) | Autogenous blood injection | 18/18 | SD rats BM-MSCs | 100μg EVs | Tail vein | 3d | 4d | NA |
| Duan 2020[2] | China | ICH | SD rats(male) | 8-9w | NA | Collagenase IV injection | 12/12 | Rat BM-MSCs | 100μg EVs | Tail vein | 1d | 1,7,28d | Beam walking test and motor deficit score |
| Liu 2021[3] | China | SAH | SD rats | 300−320 g | NA | occipital cistern secondary blood injection | 8/8 | HUC-MSC | 100 μg/mL EVs | Tail vein | NA | 1d | Self-made ratings |

**References**

1. Shen H, Yao X, Li H, Li X, Zhang T, Sun Q, Ji C, Chen G. Role of Exosomes Derived from miR-133b Modified MSCs in an Experimental Rat Model of Intracerebral Hemorrhage. J Mol Neurosci. 2018;64(3):421-30.

2. Duan S, Wang F, Cao J, Wang C. Exosomes Derived from MicroRNA-146a-5p-Enriched Bone Marrow Mesenchymal Stem Cells Alleviate Intracerebral Hemorrhage by Inhibiting Neuronal Apoptosis and Microglial M1 Polarization. Drug Des Devel Ther. 2020;14:3143-58.

3. Liu Z, Wang B, Guo Q. MiR-26b-5p-modified hUB-MSCs derived exosomes attenuate early brain injury during subarachnoid hemorrhage via MAT2A-mediated the p38 MAPK/STAT3 signaling pathway. Brain Res Bull. 2021;175:107-15.

Supplement Table S5.Methodological quality of three studies excluded studies about hemorrhagic stroke

| Author (Year) | types | peer-reviewed publication | statement of control of temperature | random allocation to treatment or control | blinded induction | blinded assessment of outcome | use of anesthetic without significant intrinsic neuroprotective activity | suitable animal models | sample size calculation | compliance with animal welfare regulations | statement of potential conflict of interests |
| --- | --- | --- | --- | --- | --- | --- | --- | --- | --- | --- | --- |
| Shen 2018[1] | ICH | √ | √ | √ | √ | √ |  | √ |  | √ | √ |
| Duan 2020[2] | ICH | √ |  | √ | √ |  |  | √ |  | √ | √ |
| Liu 2021[3] | SAH | √ | √ | √ |  |  |  | √ |  | √ | √ |

**References**

1. Shen H, Yao X, Li H, Li X, Zhang T, Sun Q, Ji C, Chen G. Role of Exosomes Derived from miR-133b Modified MSCs in an Experimental Rat Model of Intracerebral Hemorrhage. J Mol Neurosci. 2018;64(3):421-30.

2. Duan S, Wang F, Cao J, Wang C. Exosomes Derived from MicroRNA-146a-5p-Enriched Bone Marrow Mesenchymal Stem Cells Alleviate Intracerebral Hemorrhage by Inhibiting Neuronal Apoptosis and Microglial M1 Polarization. Drug Des Devel Ther. 2020;14:3143-58.

3. Liu Z, Wang B, Guo Q. MiR-26b-5p-modified hUB-MSCs derived exosomes attenuate early brain injury during subarachnoid hemorrhage via MAT2A-mediated the p38 MAPK/STAT3 signaling pathway. Brain Res Bull. 2021;175:107-15.
