# Supplementary material for: Meta-Analysis of the Therapeutic Effects of Stem Cell-Derived Extracellular Vesicles in Rodent Models of Hemorrhagic Stroke
Source: Stem Cells Int. 2024 Jun 27;2024:3390446. doi: 10.1155/2024/3390446 (PMC11390234; doi:10.1155/2024/3390446)
Supplement: Supplementary 2 — Figure 1: subgroup analysis by animal types in SAH. Figure 2: subgroup analysis by anesthetic drugs in SAH. Figure 3: subgroup analysis by methods of SAH. Figure 4: subgroup analysis by SCEVs types in SAH. Figure 5: subgroup analysis by delivery routes in SAH. Figure 6: subgroup analysis by animal types for the acute neurobehavioral scores in ICH. Figure 7: subgroup analysis by anesthetic drugs for the acute neurobehavioral scores in ICH. Figure 8: subgroup analysis by methods of ICH for the acute neurobehavioral scores. Figure 9: subgroup analysis by SCEVs types for the acute neurobehavioral score in ICH. Figure 10: subgroup analysis by animal types for the subacute neurobehavioral scores in ICH. Figure 11: subgroup analysis by anesthetic drugs for the subacute neurobehavioral scores in ICH. Figure 12: subgroup analysis by methods of ICH for the subacute neurobehavioral scores. Figure 13: subgroup analysis by animal types for the chronic neurobehavioral scores in ICH. Figure 14: subgroup analysis by anesthetic drugs for the chronic neurobehavioral scores in ICH. Figure 15: subgroup analysis by methods of ICH for the chronic neurobehavioral scores. Figure 16: subgroup analysis by SCEVs types for the chronic neurobehavioral scores in ICH. [file 3390446.f2.docx]

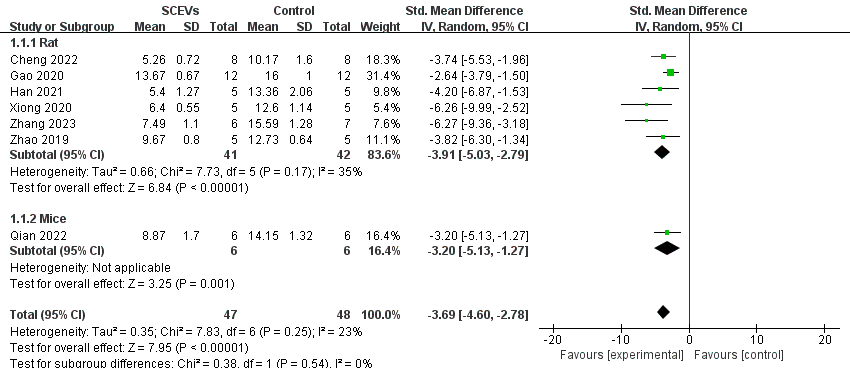


Supplement Figure S1. Subgroup analysis by animal types in SAH.


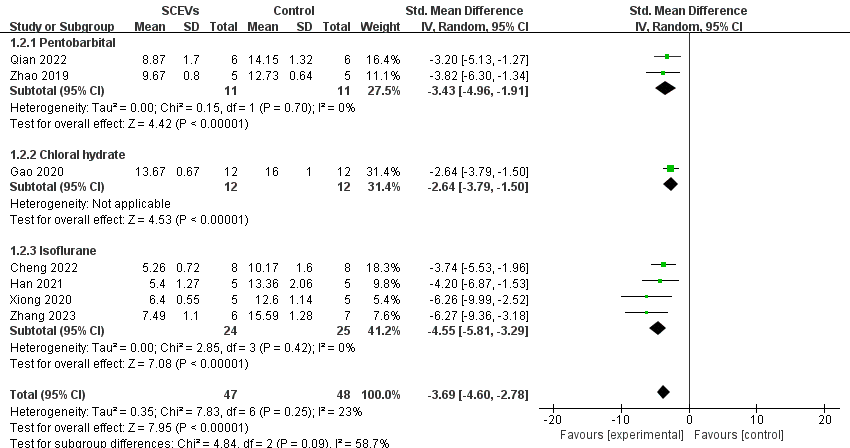


Supplement Figure S2. Subgroup analysis by anesthetic drugs in SAH.


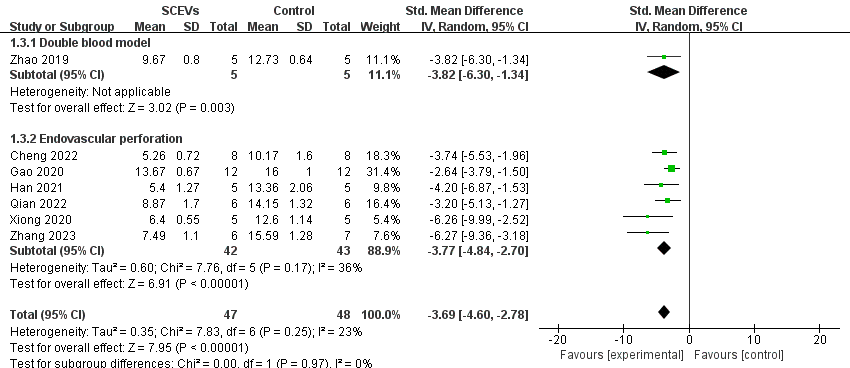


Supplement Figure S3.Subgroup analysis by methods of SAH.


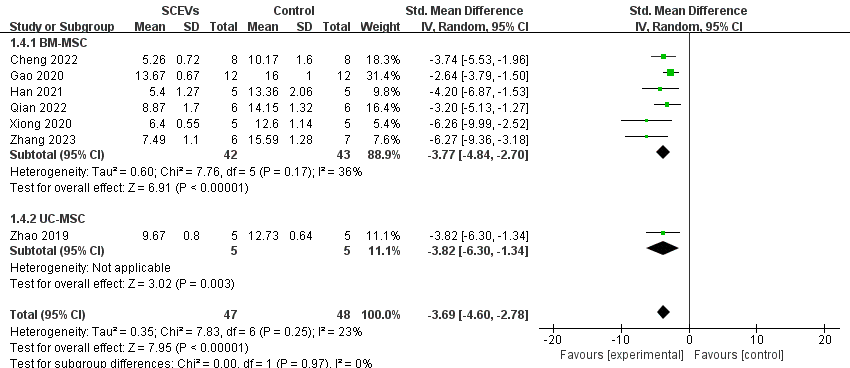


Supplement Figure S4. Subgroup analysis by SCEVs types in SAH.


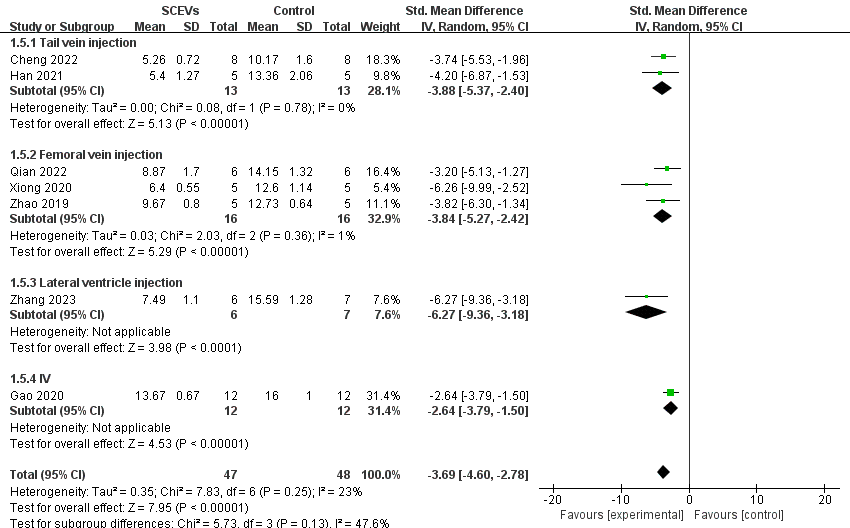


Supplement Figure S5.Subgroup analysis by delivery routes in SAH.


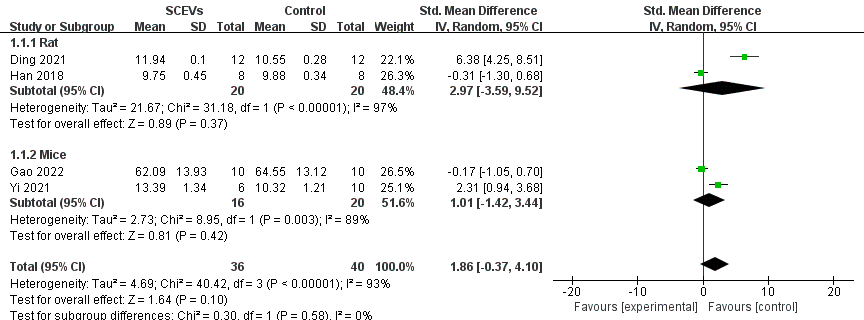


Supplement Figure S6. Subgroup analysis by animal types for the acute neurobehavioral scores in ICH.


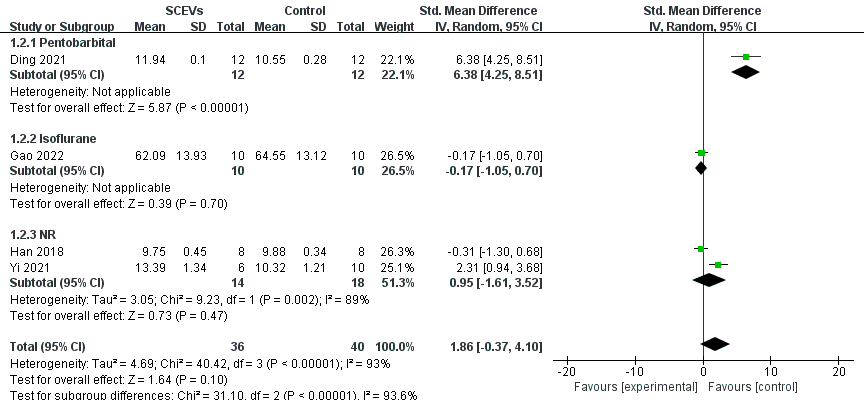


Supplement Figure S7. Subgroup analysis by anesthetic drugs for the acute neurobehavioral scores in ICH.


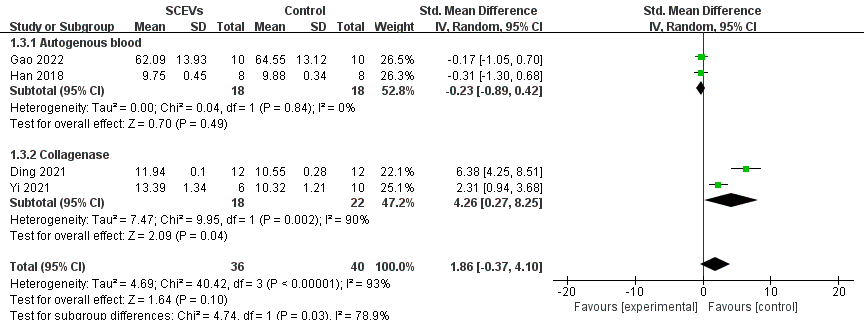


Supplement Figure S8. Subgroup analysis by methods of ICH for the acute neurobehavioral scores.


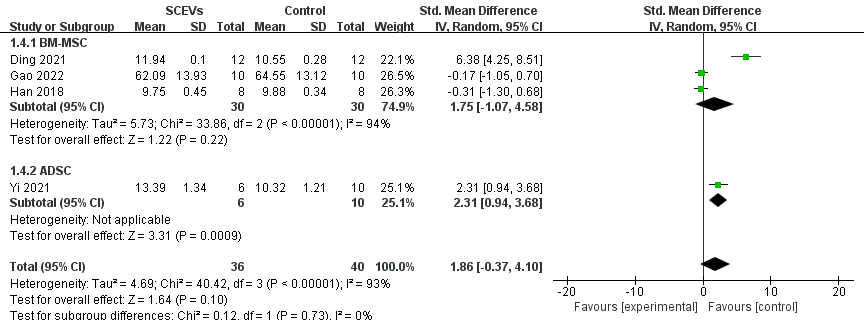


Supplement Figure S9. Subgroup analysis by SCEVs types for the acute neurobehavioral score in ICH.


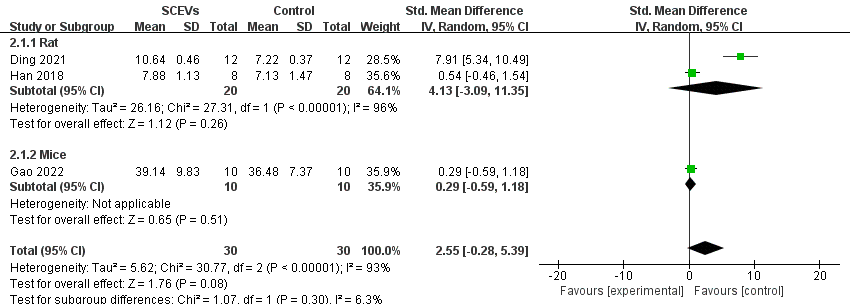


Supplement Figure S10. Subgroup analysis by animal types for the subacute neurobehavioral scores in ICH.


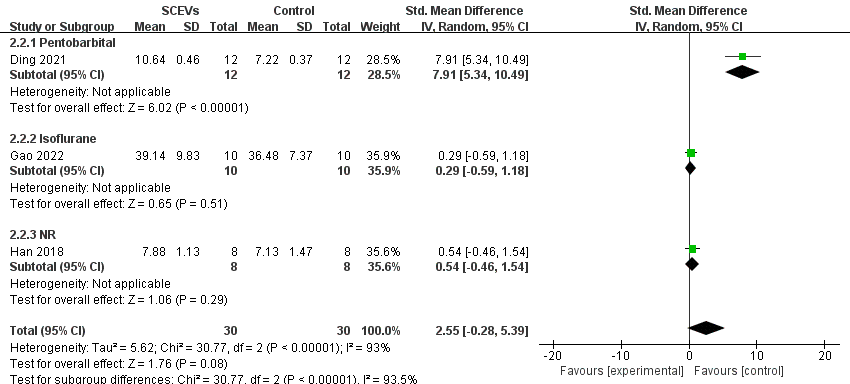


Supplement Figure S11. Subgroup analysis by anesthetic drugs for the subacute neurobehavioral scores in ICH.


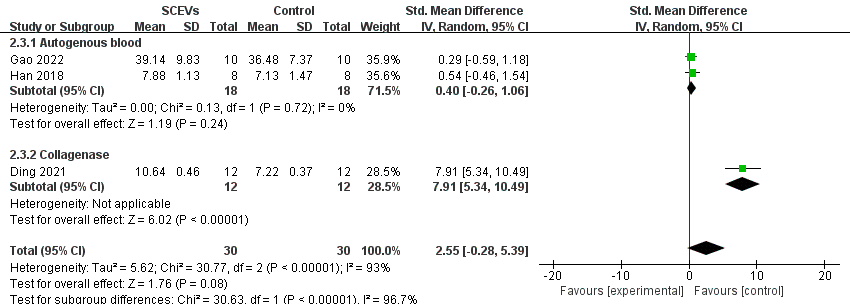


Supplement Figure S12. Subgroup analysis by methods of ICH for the subacute neurobehavioral scores.


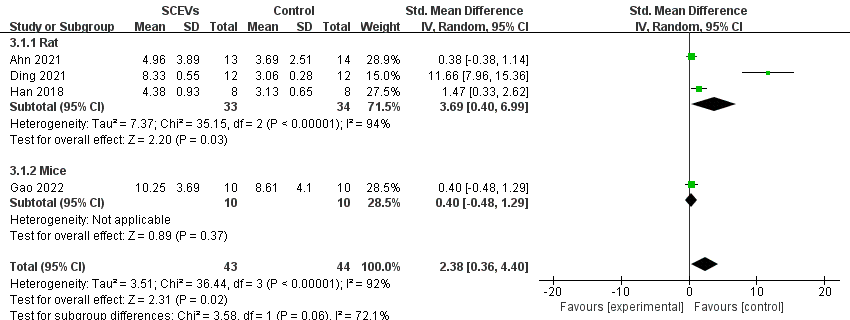


Supplement Figure S13. Subgroup analysis by animal types for the chronic neurobehavioral scores in ICH.


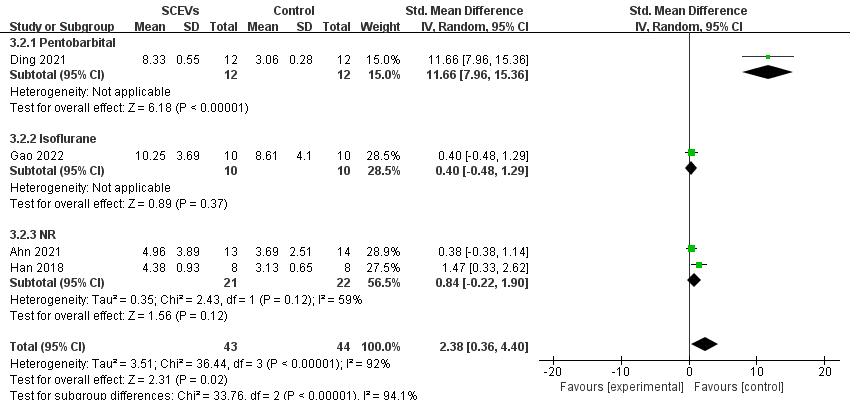


Supplement Figure S14. Subgroup analysis by anesthetic drugs for the chronic neurobehavioral scores in ICH.


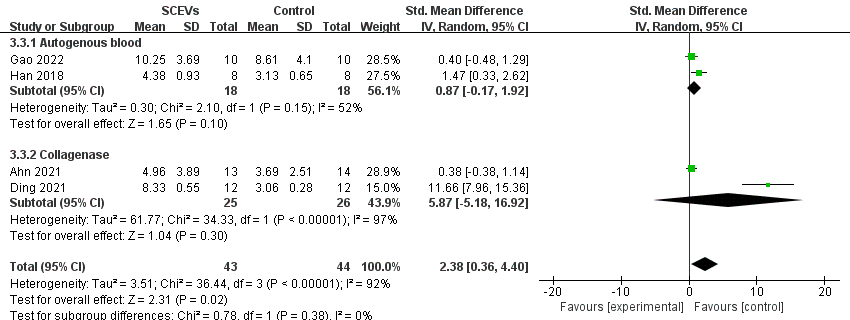


Supplement Figure S15. Subgroup analysis by methods of ICH for the chronic neurobehavioral scores.


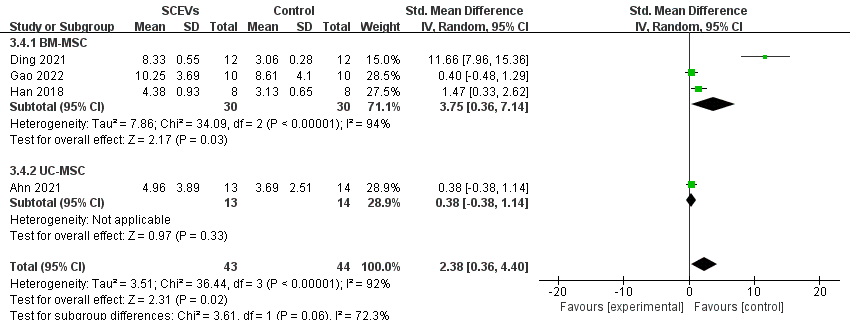


Supplement Figure S16. Subgroup analysis by SCEVs types for the chronic neurobehavioral scores in ICH.
