# Supplementary material for: Meta-Analysis of the Therapeutic Effects of Stem Cell-Derived Extracellular Vesicles in Rodent Models of Hemorrhagic Stroke
Source: Stem Cells Int. 2024 Jun 27;2024:3390446. doi: 10.1155/2024/3390446 (PMC11390234; doi:10.1155/2024/3390446)
Supplement: Supplementary 3 — Table 6: PRISMA 2020 checklist. [file 3390446.f3.docx]

| **Section and Topic** | **Item #** | **Checklist item** | **Location where item is reported** |
| --- | --- | --- | --- |
| **TITLE** | | |  |
| Title | 1 | The report is identified as a meta-analysis. | 1 |
| **ABSTRACT** | | |  |
| Abstract | 2 | The structured summary includes Background, Methods, Results and Conclusions. | 2 |
| **INTRODUCTION** | | |  |
| Rationale | 3 | Described in the Introduction section. | 4 |
| Objectives | 4 | Stated in the Introduction section. | 4 |
| **METHODS** | | |  |
| Eligibility criteria | 5 | Described in the Methods section. | 5 |
| Information sources | 6 | Registration does not apply | 5 |
| Search strategy | 7 | Described in the Methods section. | 5 |
| Selection process | 8 | Described in the Methods section. | 5 |
| Data collection process | 9 | Described in the Methods section. | 6 |
| Data items | 10a | Described in the Methods section. | 6 |
|  | 10b | Described in the Methods section. | 6 |
| Study risk of bias assessment | 11 | Described in the Methods section. | 7 |
| Effect measures | 12 | Described in the Methods section. | 7 |
| Synthesis methods | 13a | Described in the Methods section. | 7 |
|  | 13b | Described in the Methods section. | 7 |
|  | 13c | Described in the Methods section. | 7 |
|  | 13d | Described in the Methods section. | 7 |
|  | 13e | Described in the Methods section. | 7 |
|  | 13f | Described in the Methods section. | 7 |
| Reporting bias assessment | 14 | Described in the Methods section. | 7 |
| Certainty assessment | 15 | Described in the Methods section. | 7 |
| **RESULTS** | | |  |
| Study selection | 16a | Stated in the Results section. | 8 |
|  | 16b | Stated in the Results section. | 8 |
| Study characteristics | 17 | Stated in the Results section. | 8 |
| Risk of bias in studies | 18 | Stated in the Results section. | 8 |
| Results of individual studies | 19 | Stated in the Results section. | 9 |
| Results of syntheses | 20a | Stated in the Results section. | 9 |
|  | 20b | Stated in the Results section. | 9 |
|  | 20c | Stated in the Results section. | 9 |
|  | 20d | Stated in the Results section. | 9 |
| Reporting biases | 21 | Stated in the Results section. | 9 |
| Certainty of evidence | 22 | Stated in the Results section. | 10 |
| **DISCUSSION** | | |  |
| Discussion | 23a | Described in the Discussion section. | 11 |
|  | 23b | Described in the Discussion section. | 14 |
|  | 23c | Described in the Discussion section. | 14 |
|  | 23d | Described in the Discussion section. | 13 |
| **OTHER INFORMATION** | | |  |
| Registration and protocol | 24a | Registration does not apply. | None |
|  | 24b | A protocol was not prepared. | None |
|  | 24c | Registration does not apply. | None |
| Support | 25 | Described in the Funding section. | 16 |
| Competing interests | 26 | The authors declare that they have no competing interests. | 16 |
| Availability of data, code and other materials | 27 | Supplement Tables | 16 |

*From:*  Page MJ, McKenzie JE, Bossuyt PM, Boutron I, Hoffmann TC, Mulrow CD, et al. The PRISMA 2020 statement: an updated guideline for reporting systematic reviews. BMJ 2021;372:n71. doi: 10.1136/bmj.n71
